# Enhancing the decoding accuracy of EEG signals by the introduction of anchored-STFT and adversarial data augmentation method

**DOI:** 10.1038/s41598-022-07992-w

**Published:** 2022-03-10

**Authors:** Omair Ali, Muhammad Saif-ur-Rehman, Susanne Dyck, Tobias Glasmachers, Ioannis Iossifidis, Christian Klaes

**Affiliations:** 1 Faculty of Medicine, Department of Neurosurgery, University Hospital Knappschaftskrankenhaus Bochum GmbH, Bochum, Germany; 2 Institut Für Neuroinformatik, Ruhr University Bochum, Bochum, Germany; 3 Department of Computer Science, Ruhr-West University of Applied Science, Mülheim an der Ruhr, Germany; 4 Department of Electrical Engineering and Information Technology, Ruhr-University Bochum, Bochum, Germany

**Keywords:** Neural decoding, Learning algorithms

## Abstract

Brain-computer interfaces (BCIs) enable communication between humans and machines by translating brain activity into control commands. Electroencephalography (EEG) signals are one of the most used brain signals in non-invasive BCI applications but are often contaminated with noise. Therefore, it is possible that meaningful patterns for classifying EEG signals are deeply hidden. State-of-the-art deep-learning algorithms are successful in learning hidden, meaningful patterns. However, the quality and the quantity of the presented inputs are pivotal. Here, we propose a feature extraction method called anchored Short Time Fourier Transform (anchored-STFT), which is an advanced version of STFT, as it minimizes the trade-off between temporal and spectral resolution presented by STFT. In addition, we propose a data augmentation method derived from l2-norm fast gradient sign method (FGSM), called gradient norm adversarial augmentation (GNAA). GNAA is not only an augmentation method but is also used to harness adversarial inputs in EEG data, which not only improves the classification accuracy but also enhances the robustness of the classifier. In addition, we also propose a CNN architecture, namely Skip-Net, for the classification of EEG signals. The proposed pipeline outperforms the current state-of-the-art methods and yields classification accuracies of 90.7% on BCI competition II dataset III and 89.5%, 81.8%, 76.0% and 85.4%, 69.1%, 80.9% on different data distributions of BCI Competition IV dataset 2b and 2a, respectively.

## Introduction

A brain computer interface (BCI) is used to translate neural signals into command signals to control an extracorporeal robotic device^1^. Henceforth, a BCI establishes an alternative pathway of communication and control between the user and an external machine. The successful translation of neural signals into command signals is vital in the rehabilitation of physically disabled people^2–7^. The first step is to record neural signals from the areas of the brain which process the user’s intent^3,8–13^. These neural signals are recorded either by invasive^4,5^ or non-invasive methods^8,12,13^. Invasive methods include implanting electrodes in the brain at the area of interest, whereas, the most non-invasive BCI systems use EEG signals, i.e., the electrical brain activity recorded from electrodes which are placed on the scalp. In the next stage, the recorded signals are digitized and preprocessed using digital signal processors (DSPs). The preprocessed signals are then utilized to extract feature vectors, which are further fed to a decoding algorithm to finally map the given neural activity into corresponding intended actions. The output of the decoding algorithm is then transformed into control signals to control the external device.

In this study, we focus on non-invasive BCI systems using EEG signals to decode movement related information^14^. Movement related signals that are generated from the motor cortex by just imagining movements without any overt limb movement, are called motor imagery (MI)^15–17^. Classifying the MI-EEG signal is quite challenging due to two main reasons. Firstly, it has low signal-to-noise ratio. Secondly, it is a non-linear and non-stationary signal.

The successful classification of a MI-EEG signal into a corresponding control signal mainly depends on feature extraction techniques and machine learning algorithms. The current state-of-the-art feature extraction algorithms include common spatial pattern (CSP)^9,12^, short time Fourier transform (STFT)^15^ and wavelet transform (WT)^16^. The conventional classifiers used to classify EEG signals^12,18,19^ include linear discriminant analysis (LDA)^17^, Bayesian classifiers^20^ and support vector machines (SVM)^2,21^.

Recently, deep-learning algorithms produced state-of-the-art results in several computer vision tasks^22,23^. Deep learning has also gained popularity in BCI and spike sorting studies^24–26^. In^27^, a deep belief network (DBN) has outperformed SVM in the classification of MI-EEG tasks. In another study^28^, DBN was used to detect anomalies in the EEG signals. In^29^, DBN was also used to extract feature vectors for the classification algorithm. Convolution neural networks (CNNs) are also successfully used for decoding in BCI applications. In^30^, CNN was employed in classification of MI-EEG signals. To model cognitive events from EEG signals, a novel multi-dimensional feature extraction technique using recurrent convolutional neural networks was proposed in^31^.

Today, algorithms based on the CNN architecture are among the most successful algorithms in image recognition tasks. One reason behind this success is the translation invariance of CNNs. Therefore, in a few BCI studies, algorithms to convert EEG signal into an image representation are proposed. In^15^, the information about location, time, and frequency is combined using short time Fourier transform (STFT) to convert an EEG signal to an image structure. In^16^, the MI-EEG signal is transformed into an image using a wavelet transform, only later to be used by CNN for the classification of the signal. In^32^, a hybrid scale CNN architecture is presented for MI-EEG classification, which extracts the features from different frequency bands using multiple kernel scales. Furthermore, Zhang et al.^33^ reported the current state-of-the-art results for MI-EEG signals classification. Here, the authors presented a deep learning model, named EEG-Inception. EEG-Inception uses the inception layers for feature extraction. It uses the raw EEG signals as inputs and maps them to intended actions.

In this study, we presented a pipeline for MI-EEG classification, which outperformed all the current state-of-the-art studies on two publicly available datasets. The contributions of this study are as follows:

1. Conventional STFT uses a fixed-length window for the mapping of time domain signal into frequency domain, and consequently presents a trade-off between temporal and spectral resolution which is critical for feature extraction. Henceforth, an extension of short time Fourier Transform (STFT) that uses multiple windows of variable sizes for the transformation, called anchored-STFT, is proposed for better feature extraction.
2. Obtaining large, labeled data sets is still a challenge in training deep learning models for BCI applications, henceforth, a generative model-based data augmentation method called Gradient Norm adversarial augmentation (GNAA) is proposed that enhances the robustness and the classification accuracy of the classifier.
3. Since accurate predictions are critical for BCI applications, a shallow CNN-based architecture with few trainable parameters called Skip-Net is proposed which enhances the classification accuracy and avoids overfitting by adding a skip connection to a shallow architecture of CNN.

The proposed pipeline outperforms the current state-of-the-art studies by achieving the average classification accuracies of 89.5%, 81.8% and 76.0% on different data distributions of BCI Competition IV dataset 2b. It also outperforms the state-of-the-art studies of BCI Competition IV dataset 2a on its different data distributions by achieving the average classification accuracies of 85.4%, 69.1% and 80.9%.

## Materials and methods

**Figure 1:**
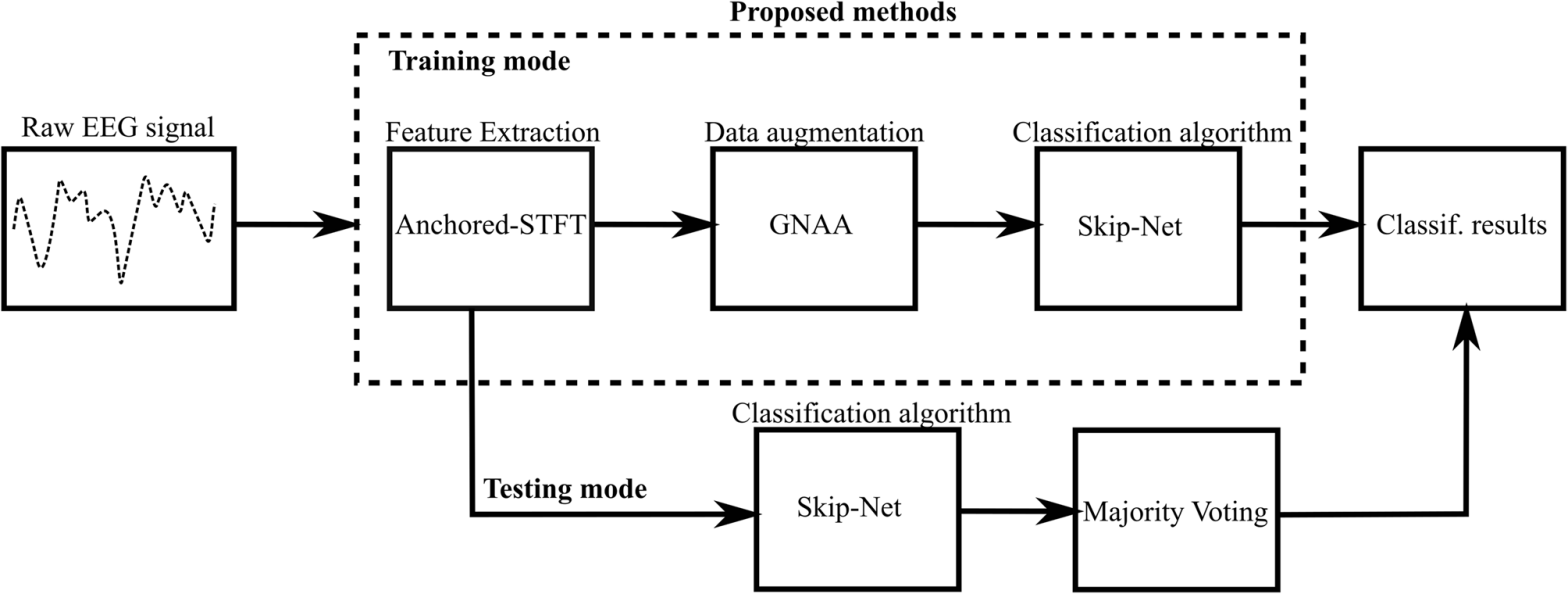
The workflow of the MI-EEG signal classification process in this study. Features are extracted from raw EEG signals using anchored-STFT. During training, the GNAA method is employed on the extracted features to generate the adversarial inputs and to enhance the amount of training data to train Skip-Net algorithm. During testing, the extracted features are directly fed to the Skip-Net algorithm to perform classification and voting is done on the output of the Skip-Net algorithm to get the final classification result.

In this study, the classification of MI-EEG signals is performed. The complete proposed pipeline of the classification process is shown as a block diagram in Fig. 1. It consists of three modules: anchored-STFT as feature extraction, GNAA as data augmentation and Skip-Net for classification. We used three publicly available datasets (BCI competition IV dataset 2a^34^, BCI competition IV dataset 2b^34^, BCI competition II dataset III^35^), which are mostly used as the benchmark for the comparison of the classification of MI-EEG signals. As we used publicly available datasets, the recording of the EEG signals is not included in the pipeline.

First, the features are extracted from EEG signals using anchored-STFT, as shown in Fig. 1. In training mode, the extracted features are then used by GNAA method to generate the adversarial inputs as well as the new legitimate training examples for the Skip-Net algorithm. In testing mode, the extracted features from the anchored-STFT are directly used by the Skip-Net algorithm for classification. Voting is done on the output of the Skip-Net algorithm to get the final classification result.

A detailed explanation of each of the modules of the pipeline is available in the following sections.

### Anchored Short-Time Fourier Transform (anchored-STFT)

Short-time Fourier transform (STFT) is a variant of Fourier transform that improves the trade-off between temporal and spectral resolution. It is used for transforming non-stationary time-series signals; signals in which the frequency components vary over time, into frequency domain. STFT extracts the segments of the time-series signal by moving a window of fixed length on the time-series signal and applies the Fourier transform on each extracted segment of the signal, hence providing time-localized frequency information of the signal. On the contrary, the standard Fourier transform considers the entire signal and results in the frequency information that is averaged over the entire time domain and consequently loses the information about the time when these frequencies occurred in the time-series signal.

Even though, STFT tries to preserve the time-localized frequency information of the signal, yet it presents a trade-off between time and frequency resolution because of a fixed-length window for the transformation of the time-series signal into frequency domain. The impact of the length of the window is directly proportional to frequency resolution and inversely proportional to time resolution. The detailed mathematical formulation of STFT can be seen in Supplementary Section ‘Short-Time Fourier Transform (STFT)’.

**Figure 2:**
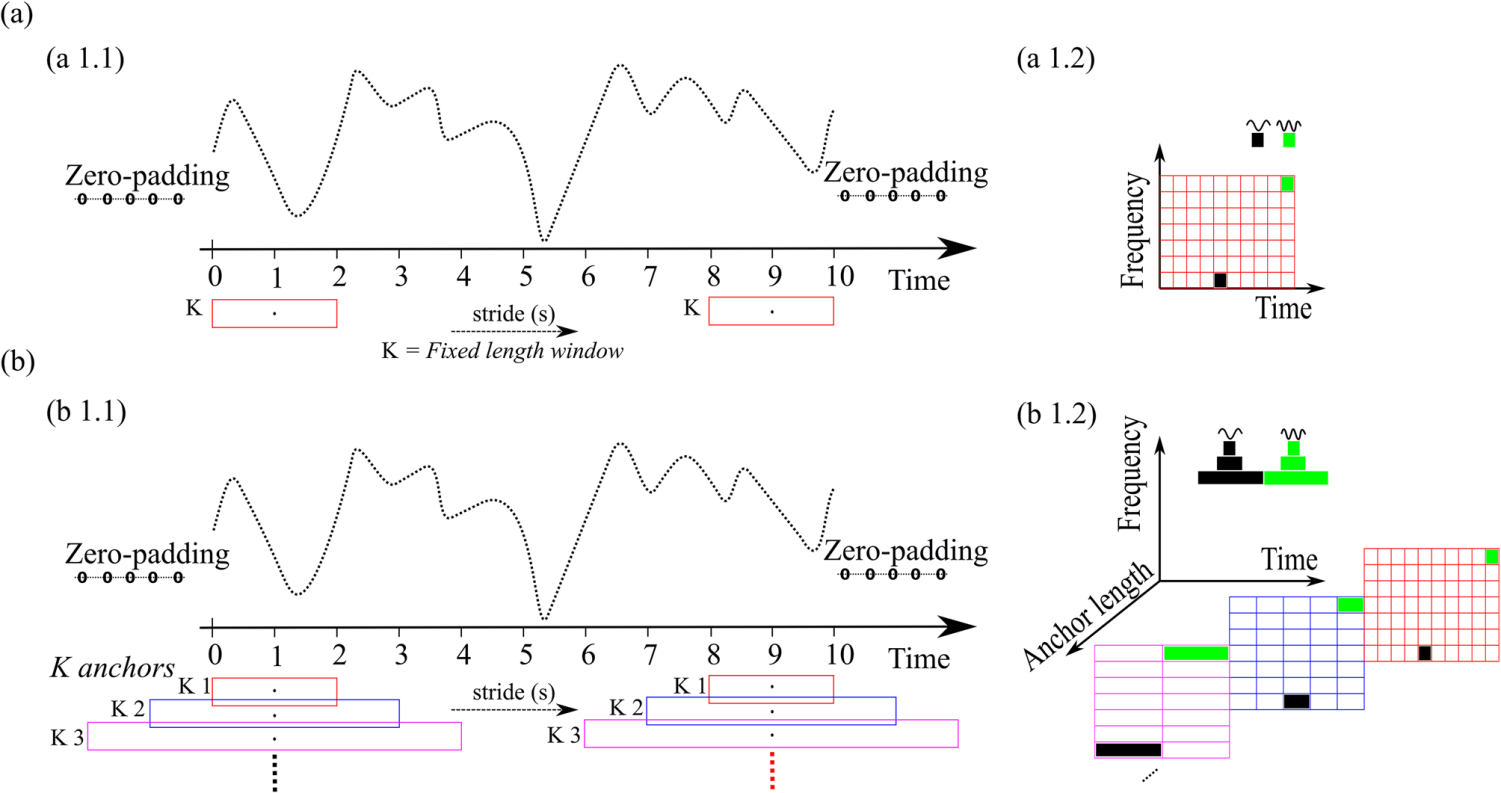
Representation of time–frequency resolution of standard STFT and anchored-STFT. (**a**) shows the time–frequency resolution of a fixed length window K of STFT. (**a 1.1**) A fixed length window K that is convolved with the time series signal with a fixed stride (s). (**a 1.2**) The spectrogram obtained by convolving the window K with time series signal. Here, frequency resolution remains the same for all locations of the spectrogram. (**b**) The time–frequency resolution of anchored-STFT. (**b 1.1**) That anchors of different lengths are convolved with the time series signal using stride (s). (**b 1.2**) That anchor K1 with short length results into better time resolution and low frequency resolution spectrogram. Anchor K3 with longer length provides better frequency but low time resolution spectrogram. The green and black colored boxes show a frequency component computed for anchors of different lengths which in turn provides different frequency resolution for each anchor length.

As STFT uses a fixed-length window (see Fig. 2(a 1.1)), the frequency resolution of the STFT remains same for all the locations in the spectrogram (see Fig. 2(a 1.2)). STFT only provides a suboptimal trade-off between time and frequency resolution. Here, an extension of STFT is proposed to address this tradeoff by defining multiple anchors of variable lengths (see Fig. 2b). The proposed algorithm is named as anchored-STFT. Anchored-STFT is inspired by wavelet transform^36^ and Faster RCNN^23^.

The working principle of anchored-STFT is as follows:

1. First, K anchors of the same shape but different lengths are defined. All the defined anchors have the same focal point (anchor position). The focal point can either be defined at the center or the left corner of the anchors (see Fig. 2(b).
2. K is the maximum number of possible anchors, which is mathematically defined in Eq. (1)
  1 $$ K = \frac{{\log \left( {sL} \right)}}{\log \left( 2 \right)} $$
3. The shape of the anchors could be selected by using the windows which are normally used by STFT e.g., Hann window etc.
  sL = length of the signal
  ${aL}^{i}$ = length of an anchor *i* = ${2}^{i}$; *i* = 1,2, …, *K*
  Minimum length of an anchor = minL = ${2}^{i=1}$
  Maximum length of an anchor = maxL = ${2}^{i=K}$
  When the focal point is defined at the centre of the anchors, then the length of the anchors is given by: ${aL}^{i}$ = length of an anchor *i* = ${2}^{i}$  + 1; *i* = 1,2, …, *K*
4. N anchors are then selected from K using grid search method, where N ⊆ K.
5. The stride ‘s’ by which the anchors are slid on time-series signal is half of the length of the anchor which has the smallest length among N selected anchors in case when the focal point is defined at the left corner of the anchors. In case when the focal point is at the center of the anchors, stride ‘s’ is defined as (minL_N ± 1)/2. minL_N = minimum length of the anchor among N selected anchors. Same stride is used for all N anchors. The length of the anchors and stride determine the number of anchor positions and consequently the number of segments of time-series signal that are extracted by the anchors.
6. Zero-padding is applied to the signal to ensure that the same amount of signal segments or frames are extracted for anchors of different lengths. Zero-padding is applied either on both ends of the signal or just one end depending on whether the anchors are centered around the anchor position or cornered at the anchor position.
7. Fourier transform is applied to each segment of the time-series signal extracted by anchors and converted to frequency domain (see Supplementary Fig. 1).
8. A separate spectrogram of the time-series signal is generated for each length anchor by aligning the spectra of adjacent, overlapping signal segments obtained by that length anchor as shown in Supplementary Fig. 1. For example, if anchors of 4 different lengths are used, then 4 spectra of the time-series signal are generated.
9. The overlap between anchors of the adjacent anchor locations and number of anchor locations are obtained by Eqs. (2) and Eq. (3), respectively.
  2 $$overlap=aL-stride$$
  3 $$no.\; of\; anchorlocations=1+\frac{sL-{minL}_{N}}{s}$$

It is clear from Fig. 2(a 1.2), that the frequency resolution of the STFT remains the same for all the locations in the spectrogram. However, it is shown in Fig. 2(b 1.2) that an anchor (K1) of smaller length provides better time resolution and lower frequency resolution, whereas the anchor (K3) of longer length provides better frequency resolution and lower time resolution. The green and black boxes show the same frequency components computed for anchors of different lengths. Each frequency component has a different resolution for each anchor of different length which consequently provides better time–frequency resolution, which is also shown in Fig. 4. Figure 4 shows the input images of different time–frequency resolution generated by 5 anchors of different lengths for right-hand MI-task performed by subject 4 of BCI competition IV dataset 2b.

An intuitive explanation of the workflow of anchored-STFT is provided in Supplementary Section ‘Workflow of anchored-STFT’.

### Gradient norm adversarial augmentation (GNAA)

In this study, we used the proposed GNAA method for harnessing new training inputs from the existing training inputs for the EEG data. The proposed data augmentation algorithm is different from any other existing data augmentation techniques. At first, it requires a trained neural network for the selection of meaningful features. Then, it calculates the gradient of cost function (of trained neural network) with respect to a given training input. We used the Frobenius norm (L^2 norm) for the normalization in Eq. (4) and Eq. (5). This gradient provides the direction of the decision boundary. The given training input $x$ is slightly perturbed (by factor $\varepsilon$) towards the direction of decision boundary. As a result, it generates new inputs ${x}_{new}$ as shown in Eq. (4). ‘Gradient norm’ method is not only a method of generating new inputs, but it also ensures the selection of features in the given feature vector that play a pivotal role in the prediction.

4 $${x}_{new}=x+\varepsilon \left(\frac{\frac{\partial \left(cost\right)}{\partial x}}{\left|\frac{\partial \left(cost\right)}{\partial x}\right|}\right)$$

We not only used Eq. (4) for data generation but also to study the existence of adversarial inputs in the domain of BCI studies. In this study, we define the term ‘adversarial inputs’ as the inputs which are modified versions of original inputs but are highly correlated. However, the employed classification algorithm fails to predict them correctly. Here, the term $\beta$ in the Eq. (5) defines the required minimum amount of perturbation, such that, the difference between two inputs (original input and perturbed input) remains indistinguishable in terms of correlation but the classifier can be fooled with perturbed inputs. The value of $\beta$ is (0.01) determined empirically.

5 $${x}_{adv}=x+\beta \left(\frac{\frac{\partial \left(cost\right)}{\partial x}}{\left|\frac{\partial \left(cost\right)}{\partial x}\right|}\right)$$

Here, we also determine the ‘pockets’ of adversarial inputs. The ‘pockets’ are defined as the number of inputs in the training dataset that can be converted into adversarial inputs (using trained classifier) by applying the amount of perturbation defined by $\beta$ in Eq. (6).

**Figure 3:**
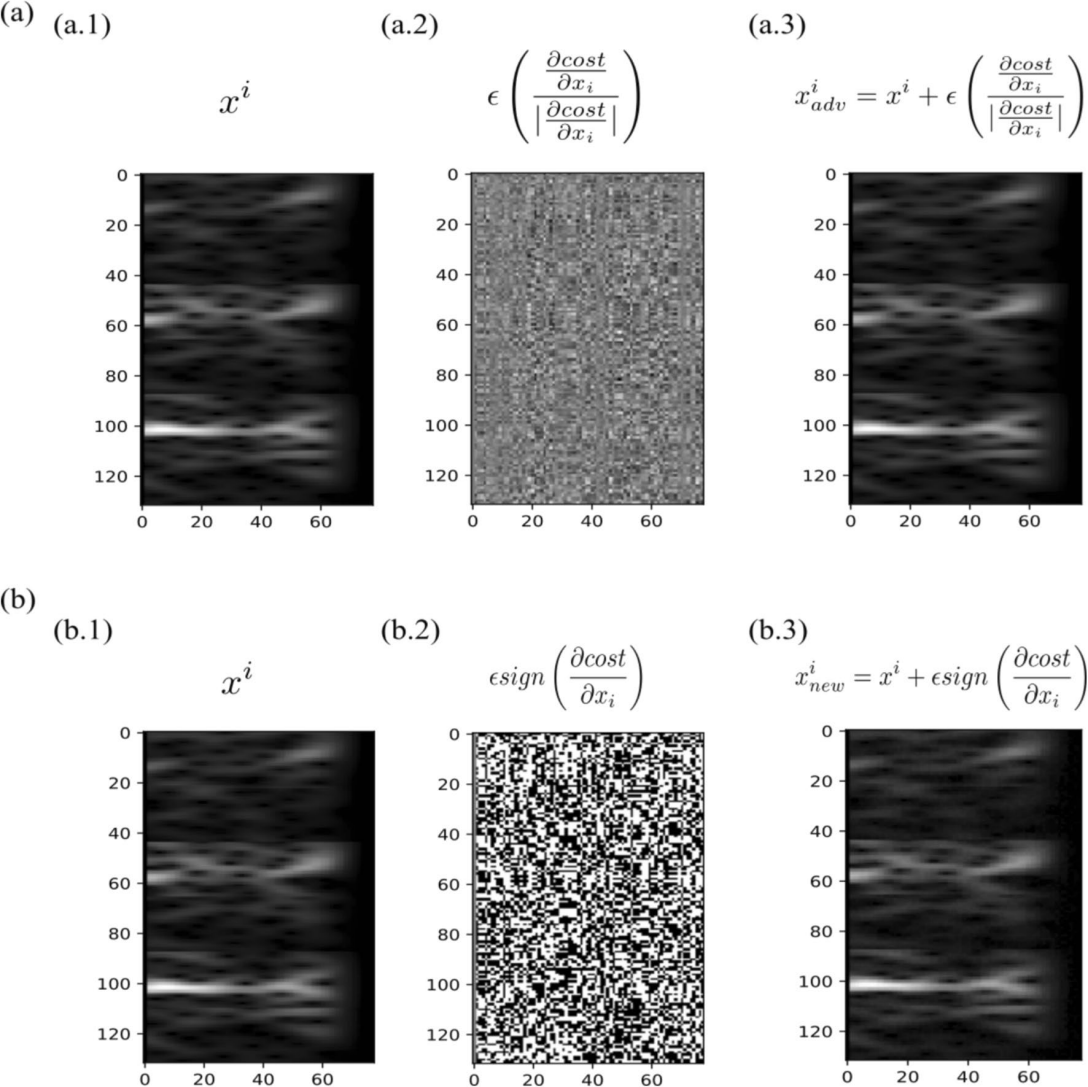
Comparison of perturbations offered by two methods; gradient norm method and gradient signum method. (**a**) The original image, perturbations produced by gradient norm method and the new generated perturbed input are shown. (**b**) The original image, perturbations produced by gradient sign method and the new generated perturbed input are shown.

Additionally, we compared the perturbation applied by the ‘gradient norm’ method with another existing method of crafting adversarial inputs called ‘gradient sign’ method^37^ defined in Eq. (6). The perturbation applied by the two methods are significantly different as shown in Fig. 3. The original input, the applied perturbation and the new generated perturbed input by the gradient norm method are shown in Fig. 3(a). Whereas the original input, the applied perturbation and the new generated perturbed input by the fast gradient sign method are shown in Fig. 3(b). The perturbation applied by the ‘gradient norm’ method carefully selects only the features that are important for the employed classification algorithm as shown in Fig. 3(a.2). The more important features are replaced with higher values and the value of the least important feature is slightly changed. The direction of the perturbations tends to be towards the decision boundary.

However, the perturbation applied by the ‘fast gradient sign’ method seems to be less informative (see Fig. 3b.2). The meaningful information in perturbation is lost because of the signum operator in Eq. (6). The signum operator maps all the values greater than 0 to 1 and the values less than 0 to − 1 in the perturbation matrix (see Fig. 3b.2). Mathematically, the signum operator is defined in Eq. (7). As a result, the perturbation matrix is filled with values of either 1 or − 1 and importance of each feature is disregarded.

6 $${x}_{adv}=x+\varepsilon sign\left(\frac{\partial \left(cost\right)}{\partial x}\right)$$

7 $$sign:=\left\{\begin{array}{c}-1\quad if\; x<0\\ 0\quad if\; x=0\\ 1\quad if\; x>0\end{array}\right.$$

### Feature formation

In this study, we used a convolutional neural network (CNN) based algorithm called Skip-Net for the classification of MI-EEG signals. Since the CNN based algorithms have shown state-of-art results in image recognition, therefore we also converted the EEG signals into images to use for classification by the Skip-Net algorithm.

In this study, three publicly available datasets (BCI competition IV dataset 2a, BCI competition IV dataset 2b, BCI competition II dataset III) are used. The data acquisition, preprocessing and the other significant details about the datasets are discussed in detail in Supplementary Section ‘Datasets & Preprocessing’. This section contains only the necessary information to extract the features from the raw EEG signals.

In case of BCI competition IV dataset 2b, the EEG signal from second 3 to second 5.5 (2.5 s in total) is considered for each trial and converted into frequency domain using anchored-STFT (see “Gradient norm adversarial augmentation (GNAA)” section). We call this interval (from second 3 to second 5.5) of the EEG signal the signal of interest (SOI) in the rest of the document. The SOI for BCI competition IV dataset 2a lasts from second 2 to second 4.5. Whereas the SOI for dataset III BCI competition II lasts from second 2.75 to second 7.25. In case of 250 Hz sampling frequency, each SOI consists of 625 samples. Anchors of five different lengths are used to transform each SOI into frequency domain. As a result, five spectrums of different time–frequency resolution are obtained for each SOI. We treat these spectra as images. The lengths (in samples) of anchors used are as follows: 16, 32, 64, 128, 256. All the lengths considered are of power of 2. Stride of 8 samples is used to slide each anchor across the SOI. Here the anchors are cornered at the anchor positions. Anchor with the shortest length (8 samples) and the stride are used to determine the number of anchor positions for all the anchors and consequently the number of segments into which each SOI is divided. This results in 78 anchor locations or segments for an SOI. Since the first anchor position considered is the first sample of the SOI, so the zero-padding is only applied after the last sample of the SOI such that the 78 segments are extracted from SOI for each anchor. Equation (8) is used to calculate the zero-padding required. 257 unique FFT points as used by^15^ are used to get the frequency components. This leads to a 257 × 78 image (spectrum) for each anchor, where 257 and 78 are the number of samples along the frequency and time axes, respectively.

8 $$Zer{o}_{padding}=stride*\left(no.\;of\;anchorlocations-1\right)-signallength+anchorlength$$

**Figure 4:**
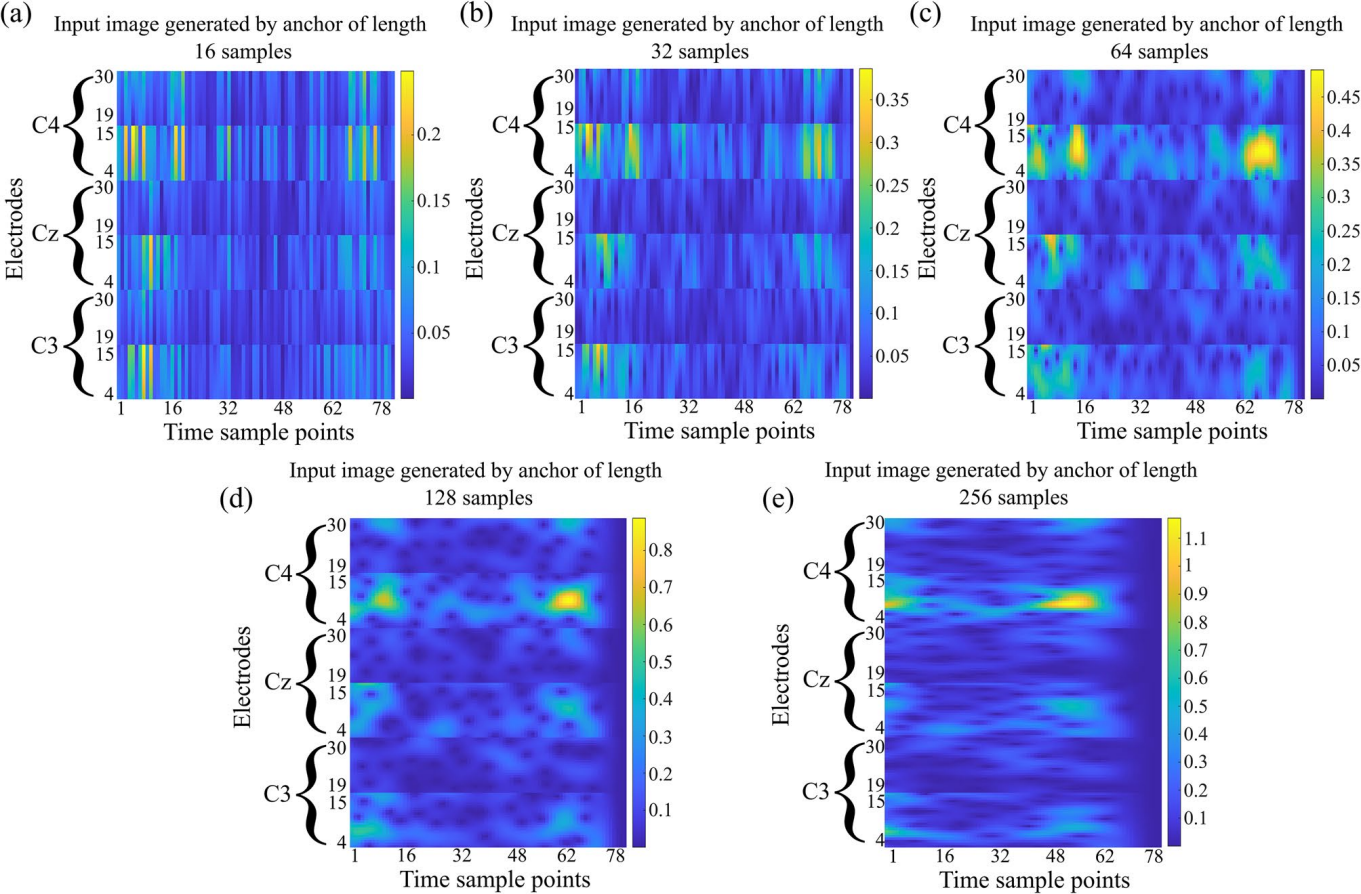
Spectral representation obtained by anchored-STFT. Input images generated by 5 anchors from an SOI of right-hand MI-task performed by subject 4.

Pfurtscheller and Lopes Da Silva^38^ showed that mu band (8–13 Hz) and beta band (13–30 Hz) are of high interest for the classification of MI-EEG signals. Since there is an event related desynchronization (ERD) and event related synchronization (ERS) in mu and beta bands respectively when an MI task is performed, these bands are vital for the classification of MI-EEG signals. Henceforth, we considered these bands for further processing. Here, the mu band is represented by frequencies between 4–15 Hz and beta band is represented by the frequencies between 19–30 Hz. We then extracted the mu and beta frequency bands from each spectrum of a SOI. The size of images for extracted mu and beta frequency bands is 22 × 78 and 23 × 78, respectively. To get the equal representation of each band, we resized the beta band to 22 × 78 using cubic interpolation method. Finally, we combined these images to get an image of size ${N}_{fr}$ x ${N}_{t}$ (44 × 78); where ${N}_{fr}$ = 44 (no. of frequency components) and ${N}_{t}$ = 78 (no. of time sample points). Since, the dataset contains the EEG signals from ${N}_{c}$ = 3 electrodes (${C}_{3}$, ${C}_{z}$ and ${C}_{4}$), we repeat the same process for all three electrodes and combine all these images from three electrodes which results in a final image of size ${N}_{h}$ × ${N}_{t}$ (132 × 78); where ${N}_{h}$ = ${N}_{fr}$ × ${N}_{c}$ = 132 for one anchor. We then repeat the whole process for all five anchors and get 5 images of size 132 × 78 each for each SOI. Figure 4 shows the input images generated by using 5 anchors for an SOI of right-hand MI-task performed by subject 4.

The decrease of energy in mu band (4–15 Hz) and increase of energy in beta band (19–30 Hz) in the C3 channel clearly shows the ERD and ERS effect, respectively for this right-hand MI-task, which is common while performing a MI-task.

Same process is done for BCI competition IV dataset 2a and dataset III of BCI competition II to get the input features.

### Skip-Net

In this study, we proposed a shallow CNN-based architecture for the classification of MI-EEG signals which contains one skip connection, hence named as Skip-Net.

The Skip-Net comprises two convolutional layers. The first convolutional layer uses filters that convolve on the time axis and extracts frequency domain features along the time axis, whereas the second convolutional layer extracts the time-domain features. We used the additive skip connection to combine the extracted frequency and time domain features to prevent the loss of any information which in turn improves the classification performance of the Skip-Net compared to other classifiers. Skip-connection enhances the classification performance. The proposed architecture contains significantly less trainable parameters as compared to its counterparts proposed in^15,29,32,33^. Skip-connection as well as less parameters also reduce the risk of overfitting.

**Figure 5:**
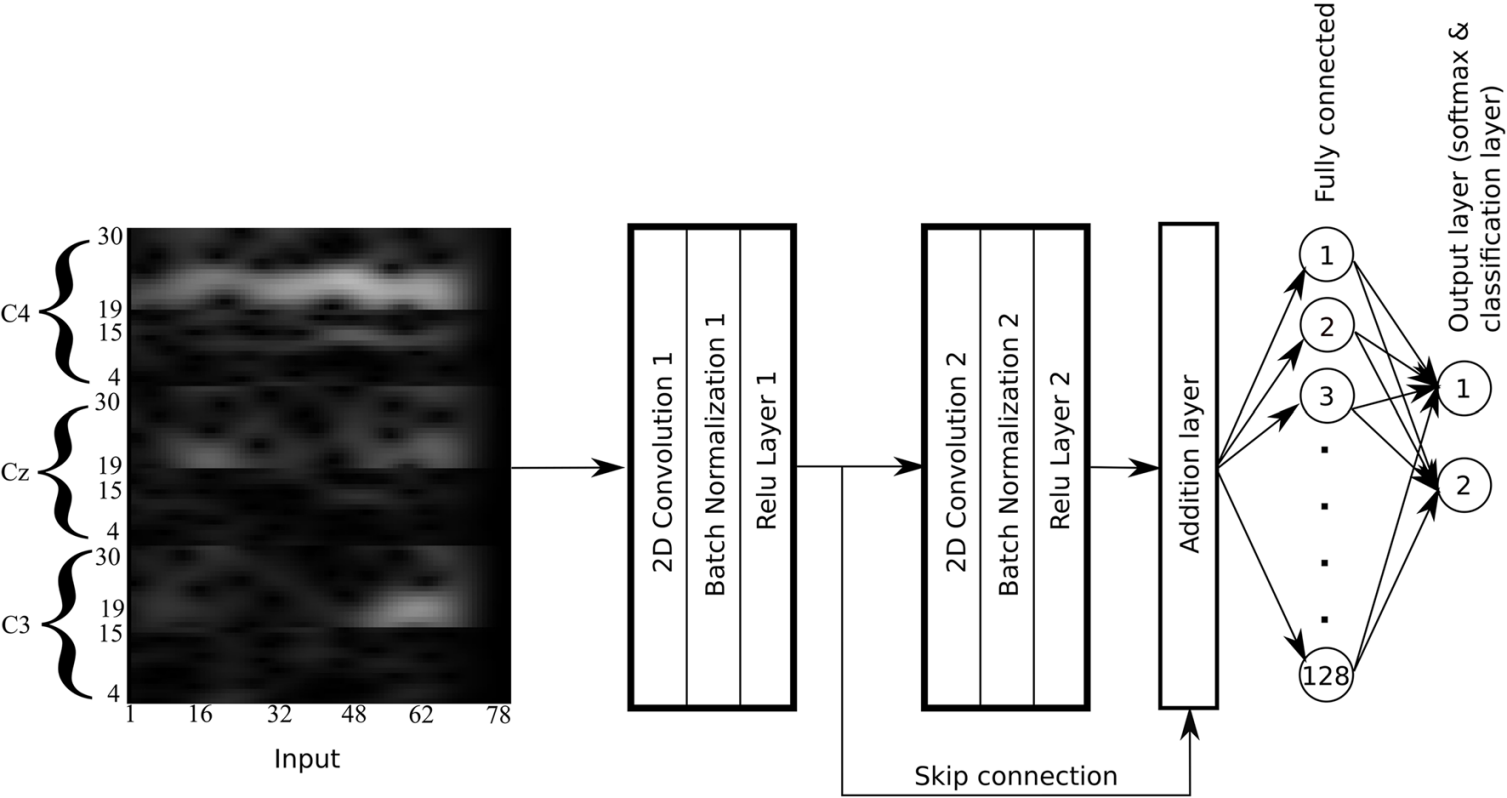
Skip-Net architecture. Illustration of the Skip-Net architecture for the classification of MI-EEG signals.

The architecture of the Skip-Net is shown in Fig. 5. First layer in Skip-Net architecture is the input layer. The dimensions of the input layer are ${N}_{h}$ × ${N}_{t}$. The second layer is the convolutional layer which uses 16 kernels of size ${N}_{h}$ × 1 to convolve the input image at a stride of 1 in both horizontal and vertical directions. Rectified linear units (ReLUs) are used as the activation functions.

The output of the convolutional layer is of the size 1 × ${N}_{t}$  × 16. Batch normalization is applied at the output of the convolutional layer. The next layer is the second convolutional layer which uses 16 kernels of size 1 × 3 to convolve the output of the last layer in horizontal direction with a stride of 1. ReLUs are used here as the activation function and batch normalization is also applied at the output of the second convolutional layer. Next layer is the addition layer which adds the output of the first ReLU and second ReLU function. Same padding is applied in the second convolutional layer to keep the dimensions of the second convolutional feature map to be the same as the output of the first convolutional feature map so that both feature maps are compatible for the addition layer. The output of the addition layer is then fed to a fully connected layer which has 128 neurons and uses a dropout of 50% as regularization to avoid overfitting. ReLUs are also used as activation function here. The last layer is the output layer, which uses Softmax function to output the predictions. The proposed architecture is inspired by residual learning framework^39^.

### Workflow at inference time

**Figure 6:**
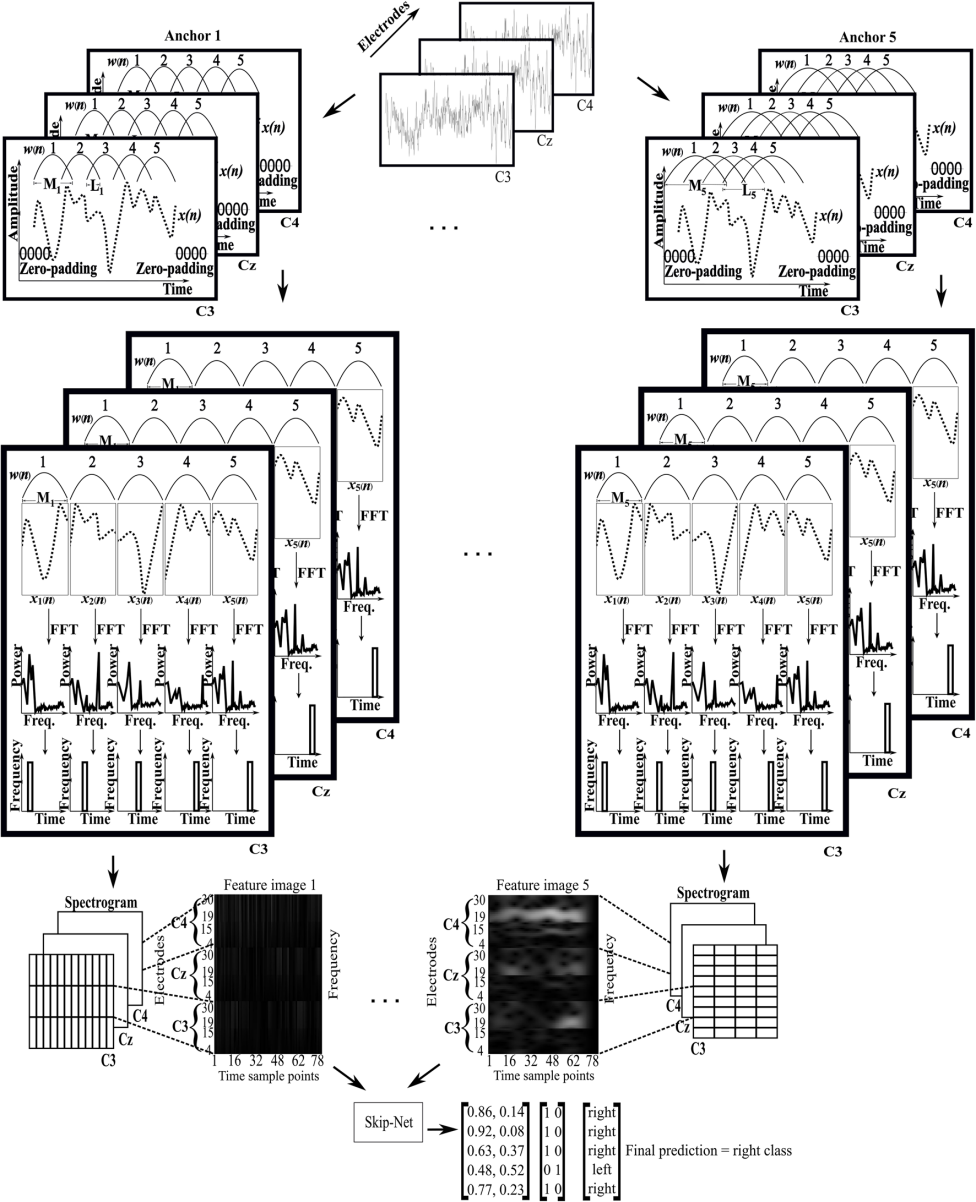
Graphical representation of whole pipeline in testing mode. Five spectra are computed for each SOI for each channel. Each spectrogram is then fed to Skip-Net to make five predictions in total for each SOI. Voting is done on five output predictions. Class with maximum number of occurrences is the final predicted class for the trial.

Figure 1 shows that the features (spectra) generated by anchored-STFT are directly used by the Skip-Net algorithm to produce the classification results in test mode. As mentioned in section Feature formation, each SOI is transformed into 5 spectra of different time–frequency resolutions as graphically represented in Fig. 6. Skip-Net classifies each spectrogram into one class which results in 5 predicted outputs for each SOI (one for each spectrogram). Final classification is based on majority voting using the 5 predicted outputs. The number of anchors (N) used must be odd to prevent ties. The graphical representation of the forward pass of the whole pipeline during the testing mode is shown in Fig. 6.

### Source code

We will upload the code and the trained models on GitHub after the successful publication of the manuscript so that others could also use it.

## Results

A detailed evaluation performance comparison of the proposed pipeline with several existing state-of-the-art studies is presented here. This section also includes the ablation studies, which briefly explains the tuning process of hyperparameters of the proposed pipeline. A comprehensive explanation of ablation study is provided in Supplementary Materials. Three publicly available, benchmark datasets are used for the validation of the results, which are as follows.

- BCI competition IV dataset 2b
- BCI competition IV dataset 2a
- BCI competition II dataset III

**BCI competition IV dataset 2b** A benchmark dataset for most of the published MI BCI studies is used with three different data distributions. This dataset contains a total of five recording sessions, 01 T, 02 T, 03 T, 04E and, 05E for each subject. Most recent studies^33^, Dai et al.^32^ first combined the data of all the available recording sessions (01 T, 02 T, 03 T, 04E, 05E), then randomly split the data into training and evaluation datasets. We named this data distribution Data-Distribution 1. Other studies^11,15,40–42^ used first three recording sessions (01 T, 02 T, 03 T) for training and last two recording sessions (04E, 05E) for the evaluation. We named this data distribution Data-Distribution 2. In addition, few studies^11^ and ^43^ also reported the cross-validation results using the first three recording sessions (01 T, 02 T, 03 T). We named this Data-Distribution 3. In this study, to have a fair comparison, the proposed pipeline is evaluated for all three data distributions, separately.

**BCI competition IV dataset 2a** Another often used benchmark dataset for the decoding of MI-tasks in BCI studies is used with three different data distributions. This dataset contains two recording sessions for each subject. The most recent studies—SW-LCR, SW-Mode in^44^, and RK-SVM in^45^—used session 1 for training and session 2 for evaluation. We named this data distribution Data-Distribution 4. DeepConvNet and EEGNet methods^46^ reported cross-validation performance in sessions 1 and 2. We named it Data-Distribution 5. MMI-LinT, MMI-nonLinT, FBCSP, CSP^47^ performed across-session analyses (training on session 1 and evaluation on session 2 and vice versa). We named it Data-Distribution 6. In addition, Ozdenizi and Erdogmus^47^ also reported cross-validation performance only in session 1. We named it Data-Distribution 7. In this study, we evaluated our pipeline for all these data distributions.

**BCI competition II dataset III** is provided with two sessions, where one session is used for training and the second session is used for the evaluation. The majority studies used the training session for training the algorithms and evaluation recording session for the validation of the result. W named this distribution as Data-Distribution 8. We also used the same criteria to validate our findings.

### Evaluation metrics

We used accuracy and kappa values as the evaluation metrics. Kappa value is calculated by Eq. (9).

9 $$kappa=\frac{accuracy-random\; accuracy}{1-random\; accuracy}$$

**Statistical analysis** We performed paired t-test to determine the statistical significance of our proposed method.

### Comparison of proposed pipeline with state-of-the-art studies on dataset 2b Competition IV

Here, the comparison of the proposed pipeline, in terms of classification accuracy and kappa values, with state-of-the-art studies^11,15,32,33,40–43,46–50^ is presented using Data-Distribution 1, Data-Distribution 2 and Data-Distribution 3.

In all the remaining analyses for all datasets and data-distributions, the values of the hyperparameters of the anchored-STFT used are as such:

- Anchors = [16,32,64,128,256].
- Stride = 8.

The details of which are provided in section Tuning of hyperparameters of anchored-STFT.

#### Comparison with state-of-the-art studies using Data-Distribution 1

In order to have a fair comparison with state-of-the-art methods using Data-Distribution 1, we restructured the data as reported in most recent study^33^. The performance comparison in shown in Table 1.

**Table 1:** Performance comparison of anchored-STFT + Skip-Net-GNAA with state-of-the-art methods on Data-Distribution 1, Data-Distribution 2 and Data-Distribution 2 of dataset 2b from BCI competition IV.

BCI IV, DATASET 2B	Method	Metric	S1	S2	S3	S4	S5	S6	S7	S8	S9	AVG
**Data-Distribution 1**	HS-CNN^32^	Acc	80.5	70.6	85.6	94.6	98.3	86.6	89.6	95.6	87.4	87.6
		Kappa	0.610	0.412	0.712	0.892	0.966	0.732	0.792	0.912	0.748	0.752
	EEG-Inception^33^	Acc	87.2	79.7	84.1	96.3	94.0	89.2	82.9	90.6	92.8	88.5
		Kappa	0.744	0.594	0.682	0.926	0.880	0.784	0.658	0.812	0.856	0.770
	EEGNet^46^	Acc	79.5	72.7	83.5	94.6	92.9	85.6	86.9	89.8	87.4	85.8
		Kappa	0.590	0.454	0.670	0.892	0.858	0.712	0.738	0.796	0.748	0.717
	**anchored-STFT + Skip-Net-GNAA**	Acc	89.8	80.4	80.4	98.2	94.6	91.7	88.5	90.6	91.7	**89.5**
		Kappa	0.796	0.608	0.608	0.964	0.892	0.834	0.770	0.812	0.834	**0.790**
**Data-Distribution 2**	EEGNet^46^	Acc	70.0	61.6	62.0	95.6	91.3	77.5	81.4	86.9	78.1	78.3
		Kappa	0.400	0.232	0.240	0.912	0.826	0.550	0.628	0.738	0.562	0.566
	TLCSD^43^	Acc	70.3	50.6	52.8	93.8	63.8	74.1	61.9	83.1	77.2	69.7
		Kappa	0.406	0.012	0.056	0.876	0.276	0.482	0.238	0.662	0.544	0.394
	RSMM^49^	Acc	72.5	56.4	55.6	97.2	88.4	78.7	77.5	91.9	83.4	77.9
		Kappa	0.450	0.128	0.112	0.944	0.768	0.574	0.550	0.838	0.668	0.558
	Bi-Spectrum ^50^	Acc	77.0	64.5	61.0	96.5	82.0	84.5	75.0	91.0	87.0	80.0
		Kappa	0.540	0.290	0.220	0.930	0.640	0.690	0.500	0.820	0.740	0.600
	CSP^11^	Acc	65.9	61.5	56.3	96.3	76.3	75.0	77.2	92.8	82.8	76.0
		Kappa	0.319	0.229	0.125	0.925	0.525	0.500	0.544	0.856	0.656	0.520
	FBCSP^11^	Acc	70.0	60.4	60.9	97.5	92.8	80.7	77.5	92.5	87.2	79.9
		Kappa	0.400	0.207	0.219	0.950	0.856	0.613	0.550	0.850	0.744	0.599
	**anchored-STFT + Skip-Net-GNAA**	Acc	75.0	61.6	59.7	96.9	92.2	87.2	81.9	93.4	87.8	**81.8**
		Kappa	0.500	0.232	0.194	0.938	0.844	0.744	0.638	0.868	0.756	**0.635**
**Data-Distribution 3**	TLCSD^43^	Acc	70.4	61.2	56.7	88.9	76.2	70.7	84.3	61.8	66.3	70.7
		Kappa	0.408	0.224	0.134	0.778	0.524	0.414	0.686	0.236	0.326	0.414
	FBCSP^11^	Acc	77.3	60.4	62.2	94.4	84.6	76.7	70.5	70.7	79.2	75.1
		Kappa	0.546	0.208	0.244	0.888	0.692	0.534	0.409	0.413	0.583	0.502
	**anchored-STFT + Skip-Net-GNAA**	Acc	79.9	57.3	56.2	95.1	87.5	83.1	75.6	71.4	77.9	**76.0**
		Kappa	0.598	0.145	0.124	0.902	0.749	0.662	0.512	0.427	0.558	**0.520**

Significant values are in bold.

**Figure 7:**
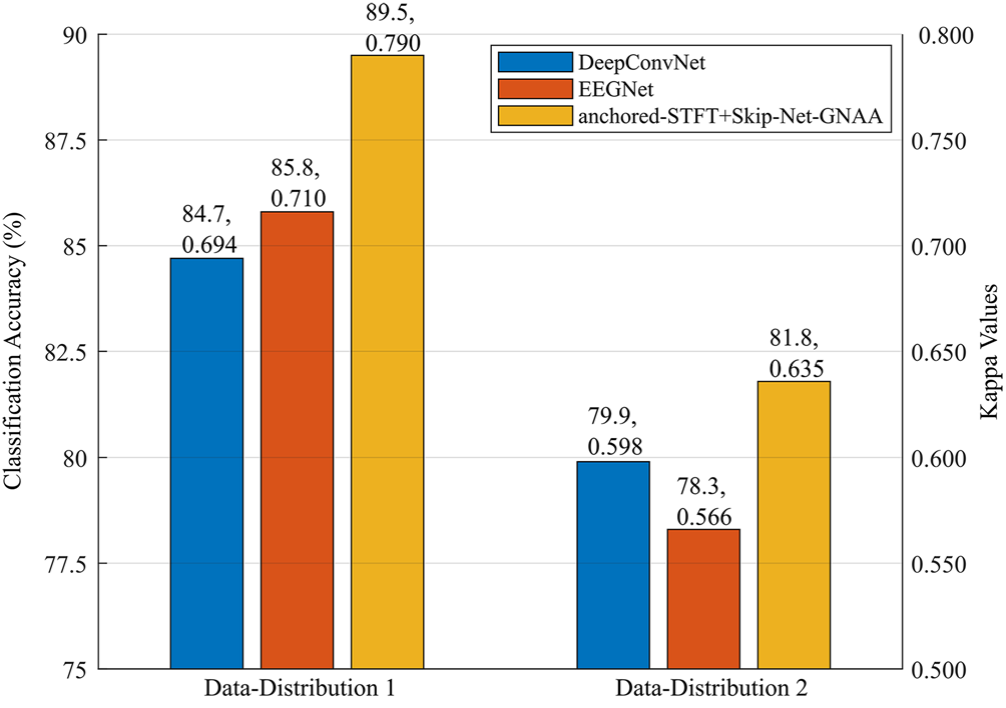
Performance comparison on dataset 2b. Performance comparison of anchored-STFT + Skip-Net-GNAA with EEGNet and DeepConvNet using Data distribution 1 and Data distribution 2 of BCI Competition IV dataset 2b.

Table 1 shows that, for Data-Distribution 1, the proposed pipeline outperformed all the state-of-the-art studies by yielding the average classification accuracy of 89.5% which is 1% higher than most recent results produced by EEG-inception method reported in^33^, whereas it is 1.9% higher than HS-CNN method reported in^32^. In addition, Fig. 7 shows the performance comparison of anchored-STFT + Skip-Net-GNAA with two well-known state-of-the-art architectures, namely EEGNet^46^ and DeepConvNet^48^ for Data-Distribution 1. It is shown in Fig. 7 that the proposed method obtained 3.7% and 4.8% higher average classification accuracy compared to EEGNet and DeepConvNet architectures, respectively. It is evident from Table 1 and Fig. 7, that the proposed method also yields the highest kappa value of 0.790, which is 5% higher than HS-CNN, whereas it is 2.6% higher than for the EEG-Inception model. These numbers are 10.1% and 13.8% for the EEGNet and DeepConvNet architectures, respectively. The proposed pipeline outperformed the EEG-inception method for 7 out of 9 subjects whereas, it outperformed HS-CNN for 6 out of 9 subjects and it outperformed EEGNet for 8 out of 9 subjects. For Data-Distribution 1, our proposed method significantly outperformed EEGNet and DeepConvNet (p < 0.05), whereas its performance is statistically similar to EEG-Inception and HS-CNN.

#### Comparison with state-of-the-art studies using Data-Distribution 2

The performance comparison of anchored-STFT + Skip-Net-GNAA with state-of-the-art methods using Data-Distribution 2 is also presented in Table 1. Table 1 illustrates that the proposed method achieved the highest average classification accuracy of 81.8%, which is 1.9% higher than the FBCSP method, whereas it is 5.8% higher than for the CSP method. These numbers are 1.8%, 3.9% and 12.1% for the Bi-Spectrum, RSMM and TLCSD methods, respectively. In addition, Fig. 7 also shows the performance comparison of anchored-STFT + Skip-Net-GNAA with EEGNet and DeepConvNet architectures for Data-Distribution 2. It is indicated from the Fig. 7, that the proposed method outperformed EEGNet by yielding an improvement of 3.5% in average classification performance whereas, it improved the accuracy by 1.9% compared to DeepConvNet. The results also indicate that anchored-STFT + Skip-Net-GNAA significantly outperformed (p < 0.05) EEGNet, TLCSD, RSMM and CSP methods, whereas it performed statistically similar to Bi-Spectrum and FBCSP algorithms.

Table 1 and Fig. 7 also imply that anchored-STFT + Skip-Net-GNAA provided the highest kappa value of 0.635 compared to other methods. It indicates that the presented method provided 22.1%, 6.0%, 5.8%, 61.1%, 13.8%, 12.2% and 6.2% improvement in terms of average kappa value with respect to CSP, FBCSP, Bi-Spectrum, TLCSD, RSMM, EEGNet and DeepConvNet methods respectively.

Table 1 shows that our method outperformed the FBCSP algorithm and Bi-Spectrum for 6 out of 9 subjects, whereas it outperformed EEGNet and RSMM for 7 out of 9 subjects and 8 out of 9 subjects, respectively. It outperformed the CSP algorithm and TLCSD for all subjects.

#### Comparison with state-of-the-art studies using Data-Distribution 3

Ang et al.^11^ introduced the Filter Bank Common Spatial Pattern (FBCSP) algorithm, which is the winner algorithm of the BCI Competition IV dataset 2b on Data-Distribution 3 and performed tenfold cross-validation on the training data. The cross-validation performance of the proposed pipeline is compared with the FBCSP algorithm and TLCSD^43^, which is shown Table 1 in terms of accuracy and kappa values.

Here, the average kappa value of the FBCSP method and TLCSD is 0.502 and 0.414 respectively, whereas the anchored-STFT + Skip-Net-GNAA obtained the average kappa value of **0.520**. The higher kappa value of the proposed methods in comparison with the other methods indicates better generalization quality. The proposed pipeline increased the kappa value by 25.6% and 3.6% with respect to TLCSD and FBCSP, respectively. Table 1 shows that the proposed approach outperformed the FBCSP method for 6 out of 9 subjects. For Data-Distribution 3, all the methods performed statistically similar.

In addition to average kappa values for tenfold cross-validation, we also compared the performance of our approach with some other methods^15,40–42^ that provided the best kappa values for dataset 2b of BCI competition IV. We also used the best kappa values of the proposed method for this comparison. Our approach outperformed the existing studies in terms of maximum kappa value comparison by yielding the maximum average kappa value of **0.737**. The detailed comparison is shown in Supplementary Table 11.

In addition, a comparison is made between the proposed pipeline and the algorithms presented in^15^. The proposed method outperformed all the presented algorithms in^15^ including its counterparts (CNN and CNN-SAE) by providing 5.6% and 2.9% higher average accuracy, respectively. The detailed comparison is given in Supplementary Table 10.

### Comparison of proposed pipeline with state-of-the-art studies on dataset 2a Competition IV

To further validate the performance of our methods, we employed our proposed pipeline on another publicly available dataset 2a from BCI competition IV. Here, we present the comparison of our algorithms with results of well-known state-of-the-art methods reported in EEGNet^46^, DeepConvNet^48^, MMI-LinT, MMI-nonLinT, FBCSP, CSP^47^, RK-SVM^45^, SW-LCR and SW-Mode^44^.

To have a fair comparison with state-of-the-art methods, we performed the same experiments using the same data distributions as used by DeepConvNet, EEGNet, MMI-LinT, MMI-nonLinT, FBCSP, CSP, RK-SVM, SW-LCR and SW-Mode methods, respectively.

The experimental protocols used by the state-of-the-art methods in respective studies are:

1. RK-SVM, SW-LCR and SW-Mode reported the performance on all possible pairwise two class MI tasks averaged over all subjects using Data-Distribution 4.
2. DeepConvNet and EEGNet methods reported four-class decoding analysis using fourfold cross-validation on Data-Distribution 5.
3. MMI-LinT, MMI-nonLinT, FBCSP, CSP performed 4-class decoding on Data-Distribution 6. In addition, 2-class (LH/RH) decoding analysis on Data-Distribution 7 is also reported for MMI-LinT, MMI-nonLinT, FBCSP, CSP.

#### Comparison with state-of-the-art studies using Data-Distribution 4

Here, a performance comparison is made between our proposed method and SW-Mode, SW-LCR, RK-SVM, SVM vec and CSP + LDA methods in pairwise MI tasks decoding. Table 2 shows the performance comparison of our proposed pipeline with other methods on all pairwise two class MI tasks averaged over all subjects. It is shown that anchored-STFT + Skip-Net-GNAA outperformed SW-Mode, SW-LCR, RK-SVM, SVM vec and CSP + LDA by yielding the highest average classification accuracy of 85.4%, which is 3.4% and 3.6% higher than SW-Mode and SW-LCR, respectively. It is 1.5% higher than RK-SVM when the geometric mean is used as the reference point, whereas these numbers are 3.2% and 3.7% when arithmetic mean and identity are used as reference point, respectively. This difference is 5.5% for CSP + LDA and 9.5% for SVM vec (half-vectorization of the covariance matrices).

**Table 2:** Performance comparison of anchored-STFT + Skip-Net-GNAA with other methods on all pairwise two class MI tasks of dataset 2a from BCI competition IV.

BCI IV, DATASET 2A	Methods		Metric	LH/RH	LH/BF	LH/TO	RH/BF	RH/TO	BF/TO	AVG
**Data-Distribution 4**	CSP + LDA^45^		Acc	75.9	80.8	82.9	84.2	81.9	73.4	79.9
			Kappa	0.518	0.616	0.658	0.684	0.638	0.468	0.598
	SVM vec^45^		Acc	73.0	78.2	81.0	77.0	77.5	68.5	75.9
			Kappa	0.460	0.564	0.620	0.540	0.550	0.370	0.518
	RK-SVM ^45^	Cref = Identity	Acc	80.6	85.0	85.0	80.5	83.6	75.5	81.7
			Kappa	0.612	0.700	0.700	0.610	0.672	0.510	0.634
		Cref = Arithmetic mean	Acc	80.6	85.8	85.6	83.6	83.5	74.2	82.2
			Kappa	0.612	0.716	0.712	0.672	0.670	0.484	0.644
		Cref = Geometric mean	Acc	79.9	87.3	86.9	85.9	86.0	77.2	83.9
			Kappa	0.598	0.746	0.738	0.718	0.720	0.544	0.678
	SW-LCR^44^		Acc	80.0	83.6	86.2	84.6	83.5	73.0	81.8
			Kappa	0.600	0.672	0.724	0.692	0.670	0.460	0.636
	SW-Mode^44^		Acc	79.8	83.7	86.0	85.0	84.0	73.4	82.0
			Kappa	0.596	0.674	0.720	0.700	0.680	0.468	0.640
	**anchored-STFT + Skip-Net-GNAA**		Acc	81.2	87.6	89.6	86.8	89.0	79.2	**85.4**
			Kappa	0.624	0.752	0.792	0.736	0.780	0.584	**0.708**

Significant values are in bold.

Similarly, anchored-STFT + Skip-Net-GNAA showed an improvement in average kappa value compared to other methods by producing the highest average kappa value of 0.708. The improvement is 10.63% and 11.3% compared to SW-Mode and SW-LCR methods. Whereas these numbers are 4.4%, 9.9% and 11.7% compared to RK-SVM with geometric mean, arithmetic mean and identity as the reference point, respectively. However, there is a significant increase of 36.7% and 18.4% compared to the SVM vec and CSP + LDA methods, respectively. For Data-Distribution 4, anchored-STFT + Skip-Net-GNAA significantly outperformed CSP + LDA, SVM vec, RK-SVM, SW-LCR and SW-Mode methods (p < 0.05).

#### Comparison with state-of-the-art studies in four-class decoding analysis using fourfold cross-validation on Data-Distribution 5

**Figure 8:**
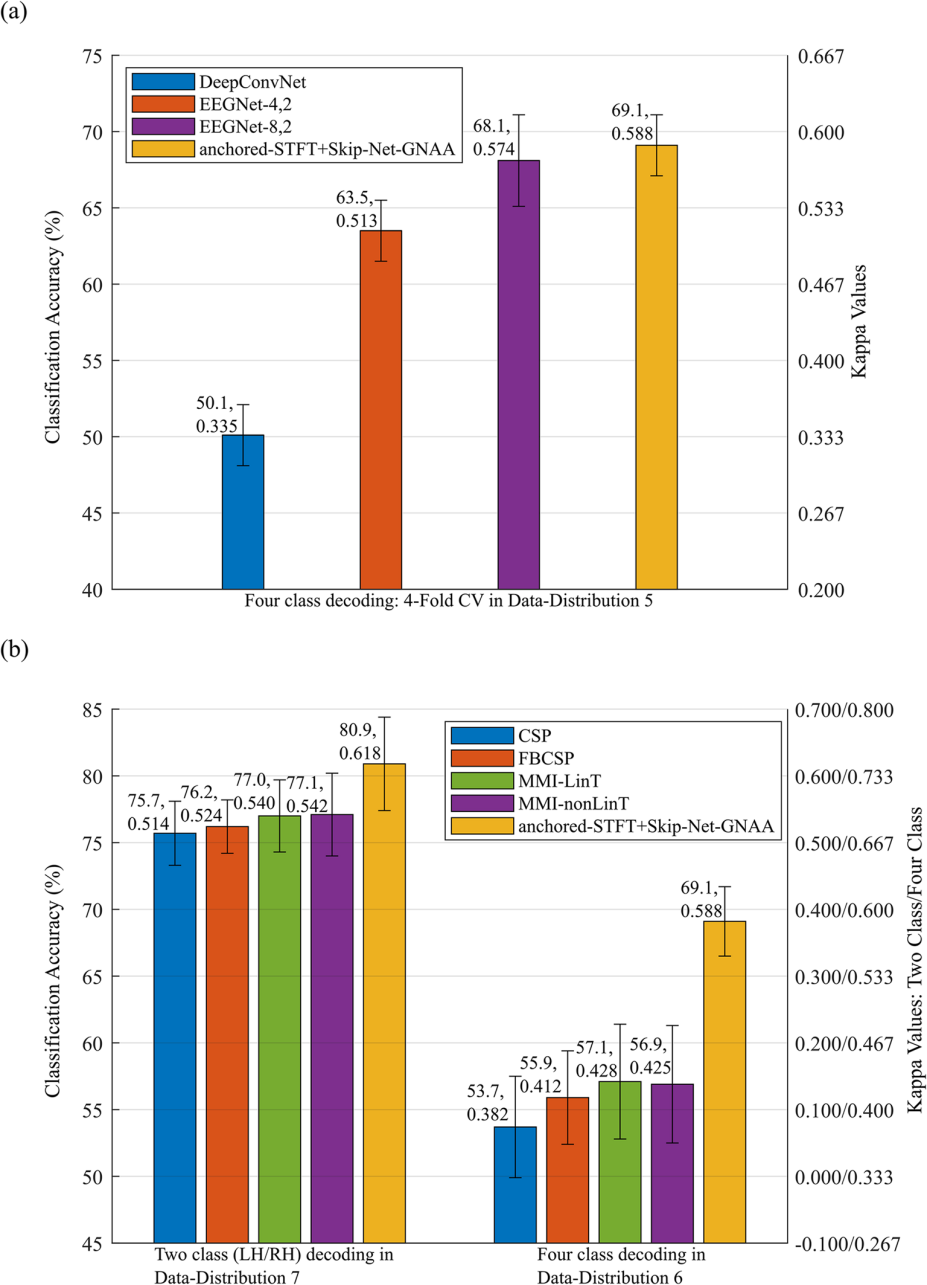
Performance comparison on dataset 2a. Performance comparison of anchored-STFT + Skip-Net-GNAA with well-known state-of-the-art methods on dataset 2a from BCI competition IV.

In addition to pairwise decoding of MI tasks, we also evaluated our pipeline in the four-class decoding protocol and compared its performance with the very well-known state-of-the-art methods i.e., EEGNet and DeepConvNet. In^46^, the authors performed fourfold cross validation in sessions 1 and 2 to evaluate the performance of the EEGNet and DeepConvNet architectures for four-class MI decoding. Henceforth, we also performed the same experiment and the performance comparison of our proposed pipeline with EEGNet and DeepConvNet is shown in Fig. 8(a). It is evident from Fig. 8(a) that anchored-STFT + Skip-Net-GNAA obtained the highest average classification accuracy as well as the highest average kappa value compared to both variants of EEGNet and DeepConvNet. The proposed method yielded an average accuracy improvement of 1% and 5.6% compared to EEGNet-8,2 and EEGNet-4,2 respectively, whereas a substantial improvement of 19% is seen compared to DeepConvNet architecture. However, these numbers are 2.4%, 14.6% and 75.5% in terms of kappa values compared to EEGNet-8,2, EEGNet-4,2 and DeepConvNet, respectively. Here, the proposed method significantly outperformed DeepConvNet and EEGNet-4,2 (p < 0.05), whereas its performance is statistically similar to EEGNet-8,2.

#### Comparison with state-of-the-art studies in four-class decoding analysis using Data-Distribution 6

An analysis is made to evaluate the performance of proposed pipeline with the state-of-the-art methods CSP, FBCSP, MMI-LinT and MMI-nonLinT in four class decoding across sessions. The performance comparison is presented in Fig. 8(b). The results in Fig. 8(b) show that anchored-STFT + Skip-Net-GNAA outperformed other methods by producing the highest average classification accuracy of 69.1% and an average kappa value of 0.588. Here, the proposed method enhanced the average classification accuracy by 15.4%, 13.2%, 12% and 12.2% compared to CSP, FBCSP, MMI-LinT and MMI-nonLinT, respectively. The same trend is also seen in terms of kappa values where the improvement is 53.9%, 42.7%, 37.4% and 38.4% compared to CSP, FBCSP, MMI-LinT and MMI-nonLinT, respectively. For Data-Distribtuion 6, the proposed pipeline significantly outperformed all the competing methods CSP, FBCSP, MMI-LinT and MMI-nonLinT (p < 0.05).

#### Comparison with state-of-the-art studies in two-class (LH/RH) decoding analysis using cross-validation on Data-Distribution 7

In addition to four-class decoding, we also performed two class decoding (left hand vs right hand) to compare the performance of our proposed pipeline with CSP, FBCSP, MMI-LinT and MMI-nonLinT. The results are presented in Fig. 8 (b). In this analysis, anchored-STFT + Skip-Net-GNAA performed the best in terms of average classification accuracy and kappa values compared to other mentioned methods. Figure 8(b) shows that anchored-STFT + Skip-Net-GNAA achieved 80.9% average classification accuracy which is 5.2% to 3.8% higher than for other methods. This range is 20.2% to 14.0% in terms of kappa values. Anchored-STFT + Skip-Net-GNAA performed significantly better than CSP, FBCSP, MMI-LinT and MMI-nonLinT (p < 0.05) for Data-Distribution 7.

### Comparison of proposed pipeline with state-of-the-art studies on BCI II, dataset III using Data-Distribution 8

To further validate the performance of our method, we employed the proposed pipeline on another publicly available dataset III from BCI competition II. Since this dataset is well divided into training and test data, the evaluation of the presented pipeline is trivial. Here, we only performed the evaluation on the unseen (test) dataset. The input images are computed as explained in the section Feature formation. Table 3 provides the comparison of classification accuracy and kappa values on this dataset produced by proposed method, methods presented in^15^ (CNN, CNN-SAE) and the winner algorithm^51^ of the BCI competition II, dataset III.

**Table 3:** Comparison of accuracy and kappa results on BCI competition II dataset III produced by anchored-STFT + Skip-Net-GNAA, CNN, CNN-SAE ^15^ and the winner algorithm ^51^.

	CNN ^15^	CNN-SAE ^15^	winner algorithm ^51^	anchored-STFT + Skip-Net-GNAA
Accuracy	89.3	90.0	89.3	**90.7**
Kappa	0.786	0.800	0.783	**0.814**

Significant values are in bold.

Table 3 illustrates that the proposed method outperformed the winner algorithm and provided 1.4% and 3.9% improvement in terms of accuracy and kappa value, respectively. It also outperformed CNN and CNN-SAE methods by 1.4% and 0.7%, respectively in terms of accuracy and 3.56% and 1.75%, respectively in terms of kappa values.

### Ablation study

BCI competition IV dataset 2b is originally provided with Data-Distribution 2 by the organizers of the competition. Using Data-Distribution 2 for tuning the parameters ensures the unfamiliarity of the evaluation data to the classifier during the training process, since there is no overlap between the two. Therefore, it is more transparent way to validate the results. Henceforth, we used data distribution 2 for the ablation study.

#### Tuning of hyperparameters of anchored-STFT

Anchored-STFT includes number, combination of anchors, and stride as the hyperparameters. The values of hyperparameters effect the evaluation accuracy as well as the computational cost. Therefore, we tried to optimize the tradeoff between evaluation accuracy and computational cost while selecting the values of the hyperparameters.

The total number of anchors in anchored-STFT are calculated using Eq. (2). In principle, a higher number of anchors results in higher classification accuracy, but that also increases the computational cost. Higher number of anchors may also increase the redundancy in the extracted information, which could cause the overfitting in shallow CNN architectures such as Skip-Net. A deeper architecture with more convolutions and fully connected layers may be required to learn the hidden meaningful patterns which in turn lead to higher computational cost, that is undesirable for online decoding of neural signals in BCI applications.

To analyze the effect of different numbers and combination of anchors on the evaluation accuracy and the computation cost, several analyses are performed which investigate the relation between the numbers and combination of anchors used and their effect on the overall evaluation accuracy and the computational power. Based on the analysis presented in Supplementary Table 1, Supplementary Table 2, Supplementary Table 3, and Supplementary Table 4 of Supplementary Materials, total number of anchors selected are 5 and the combinations used are 16,32,64,128,256.

The selection of stride is also a hyperparameter, which effects the evaluation accuracy as well as the computational cost. Stride is selected based on the anchor with smallest length. The criteria for the selection of stride are such that the overlap between smallest anchor at adjacent anchor locations is 50% minimum. However, the detail analysis of stride which results in overlap of 100%, 75%, 50%, 25% and 0% on the overall evaluation accuracy is presented in Supplementary Table 5 of Supplementary Materials. Based on the analysis, the selected stride is 8 which ensures at least the 50% overlap between the anchor of smallest length at adjacent anchor locations. This stride ensures the optimized trade-off between the evaluation accuracy and the computation cost.

In all the remaining analyses, the values of the hyperparameters used are as such:

- Anchors = [16,32,64,128,256]
- Stride = 8.

#### Performance comparison of anchored-STFT with Continuous wavelet transform (CWT) and STFT feature extraction methods and the effect of adding skip-connection to CNN architecture

Since our method is inspired from wavelet transform, and is an extension of STFT, a comparison of these methods with anchored-STFT is performed to validate the findings. Data-Distribution 2 of dataset 2b from BCI competition IV is used for this analysis. The comparison is made on two CNN based architectures i.e., proposed CNN architecture with skip connection (Skip-Net) and standard CNN architecture. Anchored-STFT using Skip-Net outperformed the STFT and CWT methods by 3.7% and 3.6%, respectively. However, the Anchored-STFT using standard CNN architecture outperformed the STFT and CWT methods by 3.1% and 5.4%, respectively. The comprehensive comparison of each subject is presented in Supplementary Table 6. This analysis depicts that adding a skip-connection to the standard CNN architecture yields improvement in the performance of the classifier.

#### Hyperparameters tuning during training for Skip-Net

The Skip-net explained in section Skip-Net is a deep-learning model. It involves several hyperparameters and the tuning of hyperparameters is done using grid search. The hyperparameters and their corresponding values after tuning used to train the Skip-Net algorithm are as follows:

1. Optimization algorithm = Adam
2. Momentum = 0.9
3. Initial Learning rate = 0.01
4. Learning rate drop factor = 0.5
5. Learning rate drop period = 5 epochs
6. Regularization = L2 norm (0.01), Dropout (0.5)
7. Max Epochs = 200
8. Mini batch size = 200

#### Evaluation of robustness of classifier using inputs generated by GNAA

It is of cardinal importance to enhance the robustness of the classifier at inference time. The data generated by GNAA improves the robustness as well as the classification accuracy. This fact is validated by a comprehensive analysis which is performed to evaluate the impact of new inputs generated by GNAA method on the robustness of the classifier. This analysis is shown in section ‘Impact of inputs generated by GNAA on robustness of classifier’ of Supplementary Materials. In addition, a quantitative comparison of perturbations generated by GNAA, and gradient sign method is performed (see section Impact of inputs generated by GNAA on robustness of classifier’ of Supplementary Materials).

The following conclusions are drawn from the aforementioned analyses:

1. The existence of adversarial inputs is not random in nature (Fig. 3a.2) as produced by gradient sign method which uses the ‘sign’ operator (see Fig. 3b.2). However, GNAA method selects only the meaningful features to perturb the inputs to generate the adversarial inputs as shown in Fig. 3(a).
2. Training the classifier on original training data plus perturbed inputs generated by GNAA method slightly improve the overall average classification accuracy as compared to gradient sign method, since the carefully perturbed inputs generate more training inputs that resemble closely the data distribution of the original training data.
3. Training the model on perturbed inputs along with the original training data enhances the robustness against adversarial attacks.
4. The perturbations applied by GNAA, and gradient sign method can provide the insight of the quality of the training data. As shown in Supplementary Table 8, subject 2 and subject 3 resulted in a greater number of adversarial examples compared to subject 4 and subject 5. It can be concluded that the discrimination power between the different classes of subject 2 and subject 3 is less as compared to subject 4 and subject 5 which is also evident from classification accuracy of these subjects as reported in Supplementary Table 7. It can also be inferred that, in case of subject 2 and subject 3, the feature vectors of distinct classes are quite close to the decision boundary determined by the classifier which also results in greater number of adversarial inputs when slightly perturbed.

## Summary and discussion

In order to enhance the quality of neural signal, we developed a novel algorithm for feature formation called anchored-STFT in conjunction with a data augmentation method named GNAA. The proposed anchored-STFT is inspired by wavelet transform^36^ and Faster RCNN^52^. Wavelets transform scales and dilates the mother wavelet. It then slides these scaled and dilated wavelets across the time-domain signal to generate a scalogram in the frequency domain. However, anchored-STFT uses anchors of different lengths. It slides these anchors across the time-domain signal to transform it to a spectrogram with different time–frequency resolution in frequency domain. Anchored-STFT generates one spectrogram for each anchor whereas the wavelet transform produces only one scalogram for all the used scales and translation factors. The anchored-STFT also addresses the limitation of standard STFT by minimizing the trade-off between temporal and spectral resolution. Anchored-STFT uses anchors of different lengths to extract segments of corresponding lengths from the time-series signal and applies Fourier transform to each extracted segmented signal. Henceforth, temporal, and spectral resolution is optimized.

The performance of deep learning algorithms is dependent on the quality as well as quantity of training examples. Therefore, in addition to feature formation technique, a data augmentation based on crafting adversarial inputs that increases the amount of training examples is proposed. The proposed data augmentation algorithm used the objective function of the previously trained model, which is trained on the original training examples. Then, the new inputs are crafted by perturbing the original training examples towards the direction of the decision boundary of the classifier. The direction of perturbation of each new input is determined by calculating the gradient of the optimized objective with respect to its original input, as defined in Eq. (5). The magnitude of the perturbation is kept small and defined by factor epsilon (see Eq. (6)).

Recently, existence of adversarial inputs in EEG based BCI studies got noticed^53,54^. Jiang and Zhang Xiao^53^ proposed transferability-based black-box attacks. At first, attacker trains a substitute model to learn the target model, and then generates adversarial inputs from the existing substitute model to attack the target model. They crafted the inputs using unsupervised fast gradient signum method (UFGSM). Contrarily, we compared the adversarial perturbations generated with original method ‘fast gradient signum method (FGSM)’ with the proposed GNAA. In FGSM and GNAA, the directions of perturbations are preserved by taking the gradient of the cost function with respect to the given input. However, the signum operator in FGSM does not keep the exact values intact. As a result, perturbations are applied with equal magnitude in all directions. On the contrary, GNAA honors the importance of each feature by generating the perturbation in each direction with different magnitude which depends on its significance.

Lastly, we proposed a shallow convolutional neural network-based architecture with a skip connection; hence, it is named Skip-Net. The Skip-Net comprises two convolutional layers. The first convolutional layer uses filters that convolve on the time axis and extracts frequency domain features along the time axis, whereas the second convolutional layer extracts the time-domain features. We used the additive skip connection to combine the extracted frequency and time domain features to prevent the loss of any information which in turn improved the classification performance of the Skip-Net compared to other classifiers.

In this study, we showed that the proposed pipeline outperformed several state-of-the-art algorithms^11,15,32,33,40–51^ on three publicly available, benchmark MI EEG datasets, as shown in Tables 1, 2, 3, Figs. 7 and 8. The aforementioned state-of-the-art studies lack uniformity in criterion to validate and compare the results. The previous studies used dataset 2a and 2b from BCI competition IV differently for the performance comparisons. Zhang et al.^33^ and Dai et al.^32^ firstly combined all the recording sessions (training and evaluation sessions) of dataset 2b and then randomly split them into training and evaluation datasets. However, Refs.^11,43,49,50^ used first three sessions of dataset 2b (training sessions) as the training dataset and the last two sessions (evaluation sessions) as the evaluation dataset as provided and recommended by the organizers of the dataset 2b from BCI competition IV. Tabar and Halici^15^ used only the training sessions of dataset 2b for the performance validation of the proposed methods. The authors used first and second recording sessions (training sessions) for training the algorithms whereas they used only the third session (third training session) for the evaluation. Similarly, different studies used dataset 2a from BCI competition IV differently for the comparison. Gaur et al.^44^, and Barachant et al.^45^ used session 1 for training and used session 2 for evaluation. However, Lawhern et al.^46^ combined all the recording sessions (training and evaluation sessions) and performed cross validation. Ozdenizi and Erdogmus^47^ performed across-session analysis (training on session 1 and evaluation on session 2 and vice versa). In addition, Ozdenizi and Erdogmus^47^ also reported cross-validation performance only in session 1. Therefore, it is very difficult to compare the proposed algorithm with previously existing algorithms. However, to overcome this important issue and to have a transparent comparison, we defined in total eight different data distributions. By defining these data distributions, the proposed algorithm is compared with existing algorithms on equal grounds. However, if the BCI community strictly follows the original data distribution of the datasets, provided by the organizer of the competition, the comparison of the algorithms can become more straight-forward, fair, and transparent. Table 1 and Fig. 7 show that the proposed method outperformed all the state-of-the-art methods such as EEG-inception, HS-CNN and EEGNet and DeepConvNet by obtaining 89.5% average classification accuracy on Data-Distribution 1 of dataset 2b from BCI competition IV. These numbers are 81.8% and 76% for Data-Distribution 2 and Data-Distribution 3 respectively. The same trend is seen in terms of kappa values. Table 2 and Fig. 8 show that the presented algorithm achieves the highest classification accuracy and kappa value 85.4% and 0.708 for Data-Distribution 4 compared to SW-Mode, SW-LCR and RK-SVM. However, for Data-Distribution 5 these numbers are 69.1% and 0.588 compared to DeepConvNet, and EEGNet. Similarly, for Data-Distribution 6 and Data-Distribution 7 it reaches to 69.1%, 0.588 and 80.9%, 0.618 respectively, compared to CSP, FBCSP, MMI-LinT and MMI-nonLinT. Table 3 shows that the proposed method outperformed the winner algorithm on Data-Distribution 8 of dataset III from BCI competition II and provided 1.4% and 3.9% improvement in terms of accuracy and kappa value, respectively. It also outperformed CNN and CNN-SAE methods by 1.4% and 0.7%, respectively in terms of accuracy and 3.56% and 1.75%, respectively in terms of kappa values. We conclude that the proposed method systematically improves the state of the art, and that in some cases the improvements are quite substantial.

The results generated by using different data distributions for training and evaluation are fairly different, as shown in Tables 1, 2, 3, Figs. 7 and 8. Therefore, in our perspective, using a standardized data distribution as provided and recommended by the organizers of the datasets would be more useful for fair comparison.

The current version of anchored-STFT constructs a separate feature matrix for each defined anchor and each feature matrix is provided to the classifier. Then, the voting strategy is applied to take the final decision. In the future, we are aiming to construct a single but more meaningful feature matrix from all the anchors. We believe that if all the necessary information is provided at once, it can increase the generalization quality of deep learning models. As a result, the computational cost of the proposed pipeline can also be reduced. Here, we briefly investigated the existence of adversarial inputs in neural data. However, more thorough investigation is required. Therefore, in future we are aiming to extract adversarial inputs created by different methods and try to train a more robust classifier by training it on data that has more variability.

## Supplementary Information

Supplementary Information.
