# Supplementary material for: Enhancing the decoding accuracy of EEG signals by the introduction of anchored-STFT and adversarial data augmentation method
Source: Sci Rep. 2022 Mar 10;12:4245. doi: 10.1038/s41598-022-07992-w (PMC8913630; doi:10.1038/s41598-022-07992-w)
Supplement: Supplementary file 1 — Supplementary Information. [file 41598_2022_7992_MOESM1_ESM.pdf]

# Enhancing the decoding accuracy of EEG signals by the introduction of anchored-STFT and adversarial data augmentation method

Omair Ali[1,4†], Muhammad Saif-ur-Rehman[3,4†], Susanne Dyck[1], Tobias Glasmachers[2], Ioannis Iossifidis[3] and Christian Klaes[1]

[1] Faculty of Medicine, Department of Neurosurgery, University hospital Knappschaftskrankenhaus Bochum GmbH, Germany, [2]Institut für Neuroinformatik, Ruhr University Bochum, Germany, [3] Department of Computer Science, Ruhr-West University of Applied Science, Mülheim an der Ruhr, Germany; [4]Department of Electrical Engineering and Information Technology, Ruhr-University Bochum

† First author (these **two authors** contributed equally.)

## Supplementary Materials

### S1. Materials and Methods

### S1.1 Short-Time Fourier Transform (STFT)

The mathematical expression of STFT is shown in equation (1).

$$X_m(\omega) = \sum_{n=-\infty}^{\infty} x(n)\, w(n - mR) e^{-j\omega n} \tag{1}$$

Where,

$x(n)$ = input signal at time *n*.

$w(n)$ = window function of length *M*.

$X_m(\omega)$ = Fast Fourier Transform of data windowed by window function $w(n)$ centered about time $mR$.

$R$ = hop size/ step size (time advance in samples).

At first, a time series signal $x(n)$ is split up into segments using a window $w(n)$ of length M. The signal in the extracted segments is tapered based on the window function used to extract the segments. Fourier transform is applied on each extracted tapered segment of the signal, and it is converted to frequency domain. Spectra of each segment of the signal is obtained which shows the strength of the frequency component with respect to time. Finally, a spectrogram is constructed by aligning the spectra of adjacent, overlapping signal segments in time-frequency plane.

### S1.2 Workflow of anchored-STFT

Workflow of anchored-STFT is shown in **Supplementary Figure 1**. In **Supplementary Figure 1**, anchors of different lengths are used to segment the time-series signal. The extracted segments of time-series signal are transformed from time domain to frequency domain. At the end, a separate spectrogram is generated for each anchor of different length. These spectra are further used by GNAA to generate augmented training data for the Skip-Net algorithm.

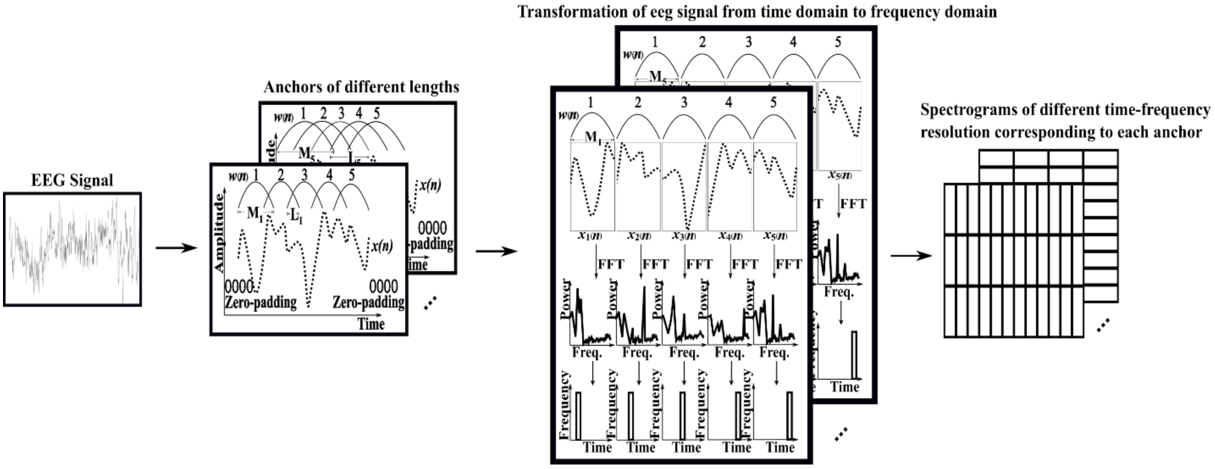


***Supplementary Figure 1:*** *Intuitive workflow of anchored-STFT. First, the anchors of different lengths are defined which are centered around or cornered at an anchor position. The anchors are then slid along the whole signal with a constant stride. Then segments of time- series signal is extracted using those anchors. Fourier transform is applied to each segment extracted by anchors and is converted to frequency domain. A Spectrogram of different time-frequency resolution is generated for each anchor which is further used as an input image by the machine learning algorithm.*

## S1.3 Difference between anchored-STFT and multitaper spectral analysis

One of the primary motivations of applying multitaper spectral analysis to time-series data is to extract the dynamics of high frequency components (such as Gamma frequency in EEG) of (very) non-time locked signals.

STFT uses fixed length windows to segment the signal and then uses a single taper (such as Hann window) to taper the signal in those fixed length windowed signals. Multitaper spectral analysis however uses multiple tapers e.g., Hann window etc. to taper the signal in those fixed length windowed signals and then apply FFT (fast fourier transform) to all the windowed tapered signals. Afterwards, it averages the power spectra corresponding to all the tapers to get a resultant average spectrum. Using multiple tapers in fixed length windows does not address the inherent time-frequency trade-off of STFT, rather it sacrifices temporal precision to enhance signal to noise ratio. The decrease in the temporal precision tends to be significant at lower frequencies. In other words, it is an extension of STFT that can be useful in low-SNR situations. Whereas anchored-STFT addresses the time-frequency trade-off by using anchors of variable lengths as explained in the manuscript section **‘2.1 Anchored Short-Time Fourier Transform (anchored-STFT)’.**

## S1.4 Datasets & Preprocessing

We used the publicly available dataset III from BCI competition II [37] and dataset 2a, 2b from BCI competition IV [34] for the evaluation of our methods, since these are the benchmark datasets for MI-EEG decoding. These datasets contain the EEG recordings from 1 and 9 subjects respectively, where each subject performed left/right hand MI tasks in dataset III from BCI competition II and dataset 2b from BCI competition IV and performed left/right hand, feet and tongue MI tasks in dataset 2a. The dataset III from BCI competition II and 2b from BCI competition IV contain the neural activity of three selected electrodes (C3, C4, Cz), which were

placed on the motor areas of the brain. Dataset 2a is recorded from 22 channels (Fz, FC3, FC1, FCz, FC2, FC4, C5, C3, C1, Cz, C2, C4, C6, CP3, CP1, CPz, CP2, CP4, P1, Pz, P2, POz). The reference electrode is located on the left mastoid. The dataset III from BCI competition II was recorded with a sampling frequency of 128 Hz whereas dataset 2a and 2b from BCI competition IV was recorded with a sampling frequency of 250 Hz and it was bandpass filtered between 0.5 Hz and 100 Hz, and a notch filter was applied at 50 Hz. BCI competition II dataset III contains 280 trials in total, out of which 140 are training trials and the remaining 140 are test trials.

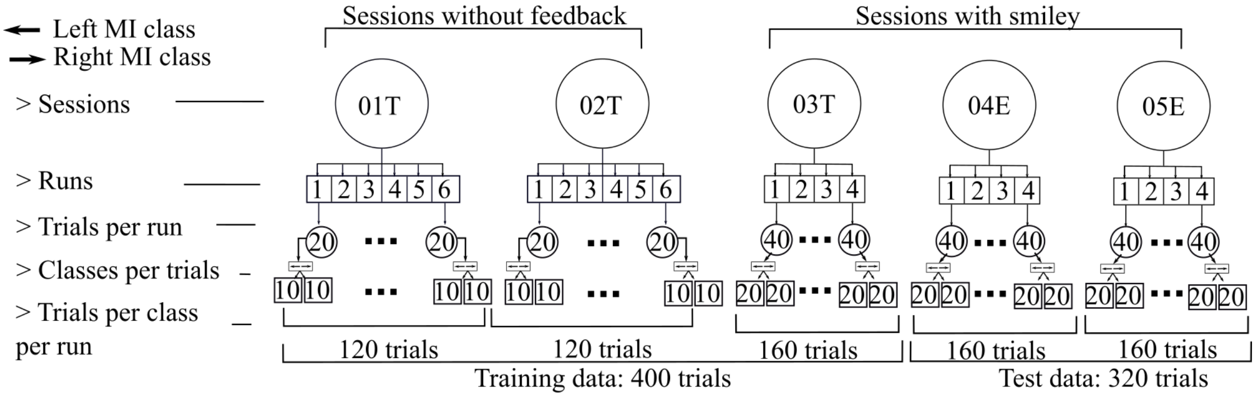


***Supplementary Figure 2:*** *The data distribution of the dataset for each subject for training and testing the algorithm. Each subject had 5 recording sessions. Session 1 and Session 2 (01T and 02T) are without feedback. Session 3, Session 4 and Session 5 (03T, 04E and 05E) are with smiley feedback. Session 1 and Session 2 had 6 runs each. Each run had 20 trials. Out of these 20 trials, 10 trials belong to left MI class and remaining10 trials belong to right MI class. Session 3, Session 4 and Session 5 had 4 runs each. Each run had 40 trails. Out of these 40 trials, 20 trials belong to left MI class and remaining 20 trials belong to right MI class.*

In BCI competition IV dataset 2a, the subjects performed 576 trials of right-hand (RH), left-hand (LH), tongue (TO) and both feet (BF) motor imagery (i.e., 144 trials per class) in two different sessions of 288 trials each, recorded on different days.

In BCI competition IV dataset 2b, five sessions were recorded for each subject, whereby first two sessions (01T and 02T) are the screening sessions without feedback, whereas the remaining sessions (03T, 04E and 05E) are online feedback sessions with smiley feedback (see **Supplementary Figure 2**). Three sessions (01T, 02T and 03T) were used for training and two sessions (04E and 05E) were used for evaluation purpose as recommended in the dataset description as shown in **Supplementary Figure 2**. The training sessions contain a total of 400 trials, out of which 200 trials belong to left MI class and the remaining 200 trials belong to right MI class. The test sessions contain a total of 320 trials for each subject. The data distribution is shown in **Supplementary Figure 2**. The experimental procedure of one trial of a screening session without feedback is shown in **Supplementary Figure 3** and that of an online feedback session with smiley feedback is shown in **Supplementary Figure 4**.

In screening sessions without feedback (see **Supplementary Figure 3**), each trial started with a fixation cross and a short acoustic alarm tone. Few seconds later, a visual cue in form of an arrow was presented for 1.25 seconds, which pointed either to the left or right based on the class. After the cue, the subjects imagined the corresponding movement for 4 seconds. At the end of each trial, a randomized intertrial interval of 1.5-2.0 seconds was added.

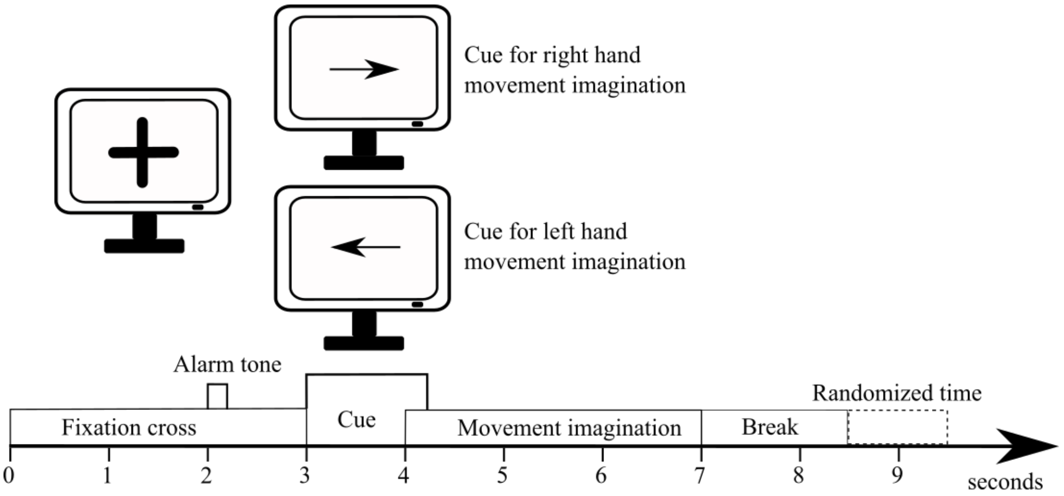


***Supplementary Figure 3**: The experimental timing scheme of one trial of screening session with no feedback. Trial began with a fixation cross on screen. Then a beep sound was given to the subjects and then at second 3, the cue was presented. From second 4 till second 7, the subjects imagined the movement based on the cue presented. This figure is modified after [34].*

In online feedback sessions with smiley feedback (see **Supplementary Figure 4**), a gray smiley was centered on the screen at the start of each trial. At second 2, a short alarm beep was given to the subject. From second 3 to second 7.5, a cue was presented and based on the cue the subjects had to imagine the corresponding movement and the classifier moved the smiley towards the direction presented by the cue. The detailed description can be found in [34]. The gray feedback smiley turned into green if it moved in the same direction as the cue, otherwise it turned into red. The screen turned black at second 7.5 which marked the end of the trail. Here, at the end of each trial an intertrial interval of 1 to 2 seconds was added.

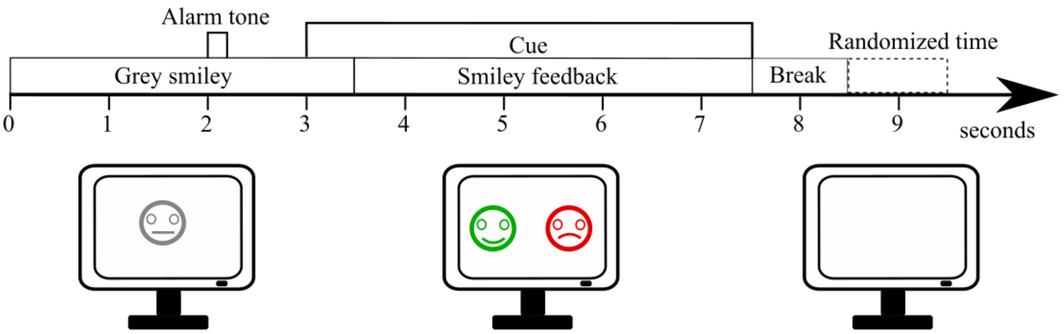


***Supplementary Figure 4:** The experimental timing scheme of one trial of online feedback session with smiley. Trial began with a grey smiley at the center of the screen. Then, a beep was given to the subjects and later from second 3 till second 7.5, a cue was presented. From second 3.5 till 7.5, subjects were supposed to imagine the movement based on the presented cue and moved the smiley in the direction of cue. Smiley turned green if it moved in the same direction as the cue, otherwise it turned red. This figure is modified after [34].*

## S2. Selection of hyperparameters of anchored-STFT

### S2.1 Effect of different numbers and different combinations of the anchors on the classification accuracy

Here, a detailed investigation of different numbers and combination of anchors is presented. Data of all nine subjects is considered to validate the selected number and combination of anchors.

### S2.1.1 Effect of single anchor of different lengths

**Supplementary Table 1** shows the evaluation performance of Skip-Net on test data using only an anchor of different lengths. It shows that single anchor of length 64 yields greater evaluation accuracy when compared to the accuracy obtained single anchors of different lengths. The evaluation accuracy shows an increasing trend from anchor of length 4 till anchor of length 64, whereas the accuracy starts decreasing afterwards.

***Supplementary Table 1:*** *Performance comparison of Skip-Net on evaluation accuracy using one anchor in anchored-STFT*

| | Anchor lengths in samples | | | | | | | |
|---|---|---|---|---|---|---|---|---|
| Subjects | 4 | 8 | 16 | 32 | 64 | 128 | 256 | 512 |
| S1 | 56.6 | 55.9 | 64.4 | 63.8 | 72.2 | 75.3 | 69.7 | 63.4 |
| S2 | 56.4 | 60.0 | 61.4 | 60.7 | 55.0 | 53.9 | 55.4 | 56.8 |
| S3 | 58.8 | 55.3 | 50.6 | 54.7 | 56.3 | 51.9 | 56.1 | 50.3 |
| S4 | 81.3 | 83.8 | 88.4 | 93.4 | 95.0 | 95.7 | 95.9 | 96.6 |
| S5 | 76.6 | 79.1 | 89.4 | 89.7 | 90.3 | 85.3 | 82.8 | 67.2 |
| S6 | 56.3 | 58.8 | 65.9 | 67.2 | 75.9 | 80.3 | 72.5 | 70.0 |
| S7 | 60.3 | 59.4 | 63.4 | 72.5 | 74.1 | 76.9 | 78.8 | 80.9 |
| S8 | 81.6 | 81.9 | 89.4 | 89.4 | 87.8 | 87.9 | 88.8 | 87.5 |
| S9 | 57.8 | 60.3 | 74.4 | 75.3 | 87.6 | 81.5 | 79.4 | 77.5 |
| **Average %** | 65.1 | 66.1 | 71.9 | 74.1 | **77.1** | 76.5 | 75.5 | 72.2 |

### S2.1.2 Effect of different combinations of three anchors

**Supplementary Table 2** shows the effect of using different combinations of three anchors on the evaluation accuracy of Skip-Net on test data. It is shown in **Supplementary Table 2**, that average classification accuracy is higher for anchors of combinations [16,32,64], [32,64,128] and [64,128,256] as compared to other combinations.

***Supplementary Table 2:*** *Performance comparison of Skip-Net on evaluation accuracy using different combinations of three anchors in anchored-STFT.*

| | Anchor combinations based on anchor lengths | | | | | |
|---|---|---|---|---|---|---|
| Subjects | [4,8,16] | [8,16,32] | [16,32,64] | [32, 64, 128] | [64,128,256] | [128,256,512] |
| S1 | 61.3 | 64.7 | 70.0 | 70.6 | 70.6 | 70.6 |
| S2 | 56.8 | 58.2 | 58.6 | 51.4 | 56.8 | 48.6 |
| S3 | 58.4 | 59.7 | 61.3 | 57.8 | 54.1 | 49.1 |
| S4 | 84.1 | 88.4 | 95.0 | 96.6 | 96.3 | 95.9 |
| S5 | 77.5 | 74.7 | 90.6 | 85.6 | 87.8 | 85.9 |
| S6 | 57.8 | 68.8 | 79.7 | 87.2 | 85.0 | 84.4 |
| S7 | 67.2 | 67.8 | 74.4 | 76.9 | 79.1 | 80.0 |
| S8 | 85.0 | 86.3 | 91.6 | 93.8 | 92.5 | 91.6 |
| S9 | 69.7 | 75.6 | 85.9 | 89.1 | 86.9 | 86.3 |
| **Average %** | 68.6 | 71.6 | 78.6 | 78.8 | **78.9** | 76.9 |

### S2.1.3 Effect of different combinations of five anchors

**Supplementary Table 3** shows the effect of using different combinations of five anchors on the evaluation accuracy of Skip-Net on test data. It is shown in **Supplementary Table 3**, anchor combination of [16,32,64,128,256] yields the highest classification accuracy on the test data compared to other combinations.

***Supplementary Table 3:*** *Performance comparison of Skip-Net on evaluation accuracy using different combinations of three anchors in anchored-STFT.*

| Subjects | [4,8,16,32, 64] | [8,16,32,64,128] | [16,32,64,128,256] | [32, 64,128,256,512] |
|---|---|---|---|---|
| S1 | 65 | 72.1 | 75.0 | 72.2 |
| S2 | 55.4 | 57.1 | 55.0 | 58.6 |
| S3 | 58.4 | 56.9 | 58.1 | 48.4 |
| S4 | 91.3 | 92.8 | 96.9 | 96.3 |
| S5 | 89.1 | 92.5 | 92.5 | 89.4 |
| S6 | 64.7 | 74.7 | 86.9 | 85.6 |
| S7 | 70.6 | 75.3 | 81.3 | 79.7 |
| S8 | 86.9 | 90.3 | 93.4 | 91.9 |
| S9 | 79.1 | 85.6 | 87.5 | 87.5 |
| **Average %** | 73.4 | 77.5 | **80.8** | 78.8 |

### S2.1.4 Effect of different combinations of seven anchors

**Supplementary Table 4** shows the effect of using different combinations of seven anchors on the evaluation accuracy of Skip-Net on test data.

***Supplementary Table 4:*** *Performance comparison of Skip-Net on evaluation accuracy using different combinations of three anchors in anchored-STFT.*

| Subjects | [4,8,16,32, 64, 128,256] | [8,16,32,64,128, 256,512] |
|---|---|---|
| S1 | 70.9 | 70.9 |
| S2 | 59.6 | 56.8 |
| S3 | 56.9 | 58.1 |
| S4 | 93.4 | 95.6 |
| S5 | 90.6 | 92.8 |
| S6 | 79.4 | 82.5 |
| S7 | 77.2 | 78.4 |
| S8 | 91.6 | 92.5 |
| S9 | 81.9 | 87.5 |
| **Average %** | 77.9 | **79.5** |

It is evident from the **Supplementary Table 1**, **Supplementary Table 2**, **Supplementary Table 3**, and **Supplementary Table 4**, that the best classification accuracy of Skip-Net on test data is obtained by five anchors of following combination [16,32,64,128,256].

### S2.2 Effect of stride on the classification accuracy

Here, an analysis is done to find the impact of different stride lengths on the classification performance of Skip-Net on the test data by employing anchored-STFT with five anchors of combination [16,32,64,128,256]. It is clear form **Supplementary Table 5**, that Skip-Net yields highest classification accuracy on test data using the five anchors mentioned above with stride of 8 which ensures 50 % minimum overlap between anchors at adjacent anchor locations. Stride of 1 yield almost 100 % overlap which generates redundant information for the Skip-Net, which is a shallow architecture, and could easily suffer from overfitting which result in decrease of the classification accuracy.

***Supplementary Table 5:*** *Effect of stride length on the classification accuracy of Skip-Net on test data.*

| | Stride length (overlap) | | | | |
|---|---|---|---|---|---|
| Subjects | 1 (~ 100 %) | 4 (~ 75 %) | 8 (~ 50%) | 12 (~ 25 %) | 16 (~ 0 %) |
| S1 | 70.60 | 69.4 | 75.0 | 71.3 | 71.3 |
| S2 | 56.8 | 57.9 | 55.0 | 60.0 | 56.1 |
| S3 | 57.2 | 61.3 | 58.1 | 57.2 | 57.8 |
| S4 | 95.6 | 96.3 | 96.9 | 95.6 | 96.3 |
| S5 | 88.8 | 92.2 | 92.5 | 88.1 | 89.7 |
| S6 | 83.1 | 86.3 | 86.9 | 85.0 | 86.3 |
| S7 | 78.4 | 79.1 | 81.3 | 78.1 | 76.3 |
| S8 | 91.9 | 91.9 | 93.4 | 92.2 | 92.5 |
| S9 | 86.9 | 85.9 | 87.5 | 85.9 | 85.2 |
| **Average** | 78.8 | 80.0 | **80.8** | 79.3 | 79.1 |

## S3. Performance comparison of anchored-STFT with Continuous wavelet transform (CWT) and STFT feature extraction methods and the effect of adding skip-connection to CNN architecture.

To validate our methods, firstly, we performed a detailed ablation study. Since our method is inspired from wavelet transform, and is an extension of STFT, a comprehensive comparison of methods is required to validate the findings regarding our proposed method. The analysis includes the performance comparison of continuous wavelet transform (CWT), STFT, and anchored-STFT as shown in **Supplementary Table 6** The comparison is made on two CNN based architectures i.e., proposed CNN architecture with skip connection (Skip-Net) and standard CNN architecture. By standard CNN architecture, we mean Skip-Net architecture (as explained in section **Skip-Net**) without the skip-connection. This analysis is required to show the effect of adding a skip-connection in the standard CNN architecture on the performance of neural signal decoding. Dataset 2b of BCI competition IV is used for this analysis. In this analysis, training sessions (01T, 02T and 03T) are used for training the classifier whereas, test sessions (04E and 05E) are used for the evaluation.

**Supplementary Table 6** shows that adding skip-connection to standard CNN architecture yields an improvement in classification performance for all three feature extraction methods (CWT, STFT and anchored-STFT). However anchored-STFT in combination with Skip-Net outperformed the CWT and STFT by 3.6 % and 3.7 % respectively.

***Supplementary Table 6:*** *Performance comparison of CWT, STFT and anchored-STFT on dataset 2b of BCI competition IV using Skip-Net and Standard CNN architectures.*

| | Standard CNN (Evaluation accuracy in %) | | | Skip-Net (Evaluation accuracy in %) | | |
|---|---|---|---|---|---|---|
| Subjects | CWT | STFT | Anchored-STFT | CWT | STFT | Anchored-STFT |
| S1 | 70.6 | 69.7 | 72.8 | 74.4 | 72.2 | 75.0 |
| S2 | 55.1 | 53.9 | 57.4 | 59.8 | 55.0 | 55.0 |
| S3 | 53.4 | 59.4 | 57.8 | 54.1 | 56.3 | 58.1 |
| S4 | 95.3 | 95.6 | 96.6 | 96.3 | 95.0 | 96.9 |
| S5 | 80.8 | 88.1 | 91.2 | 84.7 | 90.3 | 92.5 |
| S6 | 73.4 | 80.8 | 87.8 | 75.9 | 75.9 | 86.9 |
| S7 | 70.6 | 72.5 | 77.5 | 76.3 | 74.1 | 81.3 |
| S8 | 87.5 | 86.3 | 91.9 | 91.3 | 87.8 | 93.4 |
| S9 | 81.7 | 83.4 | 84.1 | 82.8 | 87.6 | 87.5 |
| **Average** | 74.3 | 76.6 | **79.7** | 77.2 | 77.1 | **80.8** |

For CWT, 'Gabor wavelet' is used as the mother wavelet. The frequency limits are kept between 1 Hz and 50 Hz. As a result, a scalogram is obtained which is then used to extract the information in the same mu and beta frequency ranges as used for STFT and anchored-STFT methods. The extracted information in mu and beta frequency ranges are resized using cubic interpolation method to achieve the same frequency dimension of input image (132) as for STFT and anchored-STFT methods, whereas the time dimension is equal to the length of the SOI.

Feature formation for STFT is mentioned in [15] and anchored-STFT is mentioned in section **Feature formation**.

## S4. Impact of inputs generated by GNAA on robustness of classifier

In this analysis, a comparison is made to evaluate the robustness of the trained model at the inference time. This analysis also shows the effect of training the model on the inputs generated by GNAA along with original training data on the overall average classification performance. For this analysis, we used dataset 2b from BCI competition IV. First three sessions (01T,02T and 03T) are used as training data (X) whereas last two sessions (04E and 05E) are used as test data (Y). The process of generating adversarial inputs and its evaluation is as follows:

- In the first step, trained anchored-STFT based Skip-Net model is used to generate the perturbed examples for the only correctly classified test inputs using both the GNAA and gradient Sign method as mentioned in section **Gradient Norm Adversarial Augmentation (GNAA)**. **Supplementary Figure 5** (a) and **Supplementary Table 7** show the graphical representation of the evaluation of Skip-Net and its performance on test data (Y) respectively. **Supplementary Figure 5**(b) shows the graphical representation of crafting perturbed inputs from the correctly classified test inputs (Y_corr) using GNAA and gradient sign method.

***Supplementary Table 7:*** *Performance of Skip-Net on test data (Y)*

| Subjects | Accuracy (%) (Y_corr) |
|---|---|
| S1 | 75.0 |
| S2 | 55.0 |
| S3 | 58.1 |
| S4 | 96.9 |
| S5 | 92.5 |
| S6 | 86.9 |
| S7 | 81.3 |
| S8 | 93.4 |
| S9 | 87.5 |
| **Average** | **80.8** |

(a)

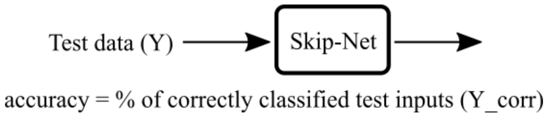


(b)

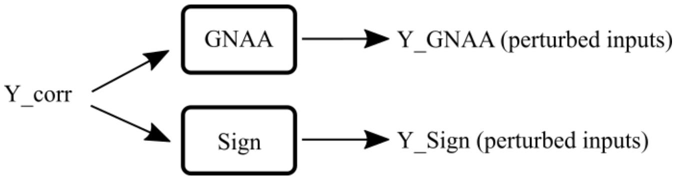


***Supplementary Figure 5:*** *Graphical representation of generation of perturbed inputs from test data using GNAA and gradient Sign methods.*

- In second step, the perturbed inputs (Y_GNAA, Y_Sign) generated in step 1 are used to evaluate the trained Skip-Net model. The performance of Skip-Net against adversarial attack (GNAA, gradient Sign method) is shown in **Supplementary Table 8**. It is evident from **Supplementary Table 8**, that on average 17.2 % and 17.1 % of perturbed inputs (Y_Sign and Y_GNAA respectively) become adversarial inputs and successfully fool the Skip-Net model.

***Supplementary Table 8:*** *Performance of Skip-Net against adversarial attack*

| | % Correctly classified after perturbation, (adversarial inputs) | |
|---|---|---|
| Subjects | Y_Sign | Y_GNAA |
| S1 | 79.2, (20.8) | 78.8, (21.2) |
| S2 | 52.2, (47.8) | 54.1, (45.9) |
| S3 | 53.6, (46.4) | 53.1, (46.9) |
| S4 | 97.2, (2.8) | 97.0, (3.0) |
| S5 | 96.1, (3.9) | 95.6, (4.4) |
| S6 | 90.2, (9.8) | 90.5, (9.5) |
| S7 | 87.9, (12.1) | 88.6, (11.4) |
| S8 | 96.2, (3.8) | 96.4, (3.6) |
| S9 | 92.6, (7.4) | 92.4, (7.6) |
| **Average** | **82.8, (17.2)** | **82.9, (17.1)** |

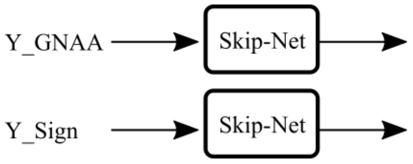


***Supplementary Figure 6:*** *Evaluation of Skip-Net against adversarial attacks when it is only trained on the original training data.*

- In the third step, the correctly classified training inputs are perturbed using both the GNAA and gradient sign methods to generate the new training examples X_GNAA and X_Sign, respectively

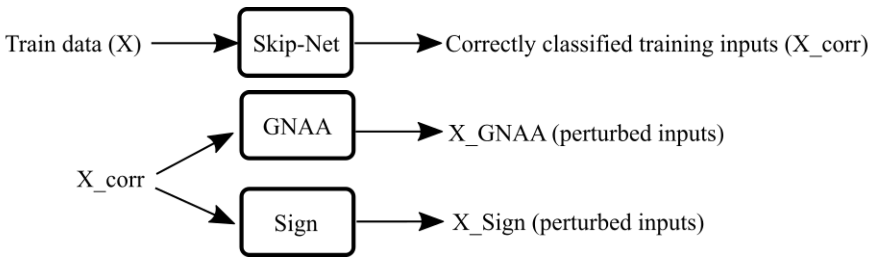


***Supplementary Figure 7:*** *Generation of perturbed inputs from correctly classified training inputs.*

- In the fourth step, the original training data, and the perturbed inputs (X_GNAA) generated in step 3 are combined to retrain the Skip-Net model which is named as 'Skip-Net-GNAA' whereas, the original training data and the perturbed inputs (X_Sign) generated in step 3 are combined together to retrain a separate Skip-Net model which is named as 'Skip-Net-Sign.

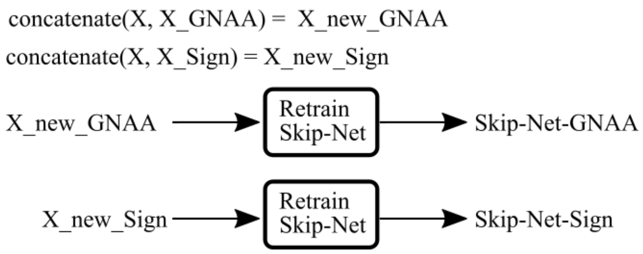


***Supplementary Figure 8:*** *Retraining of Skip-Net on original training data and perturbed inputs generated by GNAA and gradient Sign methods, which results into Skip-Net-GNAA and Skip-Net-Sign models, respectively.*

*In the fifth step, Skip-Net-GNAA, which is now trained on the enhanced training data, is evaluated for its robustness, and is shown in*

***Supplementary Table 9***. *Additionally, the impact of enhanced training dataset on the evaluation performance on original test data (Y) is reported in*

- **Supplementary Table 9**. Same analysis is performed for Skip-Net-Sign model.
- 
- **Supplementary Table 9** shows that training the Skip-Net on the enhanced training dataset not only results in enhanced robustness against adversarial attacks but also improves the overall average classification accuracy. Skip-Net-GNAA yields in improvement of classification accuracy by 1 %, whereas Skip-Net-Sign improves it by 0.3 %.

*Supplementary Table 9: Performance of Skip-Net-GNAA and Skip-Net-Sign and their performance on test data (Y)*

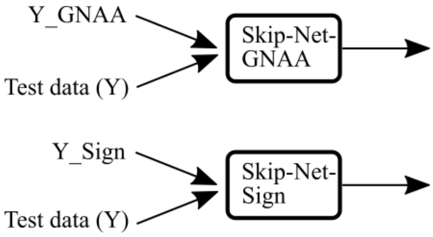


| | % Correctly classified after perturbation, (adversarial inputs) | | Test data (Y) | |
|---|---|---|---|---|
| Subjects | Y_Sign | Y_GNAA | Skip-Net-Sign | Skip-Net-GNAA |
| S1 | 79.3, (20.7) | 80.2, (19.8) | 75.0 | 75.0 |
| S2 | 59.3, (40.7) | 62.6, (37.4) | 61.0 | 61.6 |
| S3 | 79.7, (20.3) | 76.6, (23.4) | 60.6 | 59.7 |
| S4 | 98.0, (2) | 98.2, (1.8) | 96.9 | 96.9 |
| S5 | 96.2, (3.8) | 96.5, (3.5) | 92.2 | 91.2 |
| S6 | 90.9, (9.1) | 91.2, (8.8) | 86.5 | 87.2 |
| S7 | 88.1, (11.9) | 91.1, (8.9) | 77.3 | 81.9 |
| S8 | 96.4, (3.6) | 96.5, (3.5) | 93.1 | 93.4 |
| S9 | 92.9, (7.1) | 93.2, (6.8) | 86.9 | 87.8 |
| **Average** | **86.7, (13.3)** | **87.3, (12.7)** | **81.1** | **81.8** |

***Supplementary Figure 9**: Performance comparison of Skip-Net-Sign and Skip-Net-GNAA on original test data (Y) as well as robustness.*

## S5. Comparison with state-of-the-art studies

### S5.1 Session-to-session classification performance in comparison with [15]

[15] used STFT for feature vector extraction and employed deep-learning architectures for classification which includes CNN, stacked autoencoder (SAE) and CNN in conjunction with stacked autoencoder (CNN-SAE). Here, they used the first two training sessions (01T and 02T) for training the algorithms and the remaining third session (03T) for evaluation. They used accuracy results as the performance metrics. Henceforth, we also used the same data for training and evaluation and same performance metric for comparison of our proposed pipeline in this analysis.

**Supplementary Table 10** shows the comparison of the evaluation accuracy of the proposed method (anchored-STFT + Skip-Net-GNAA) with CNN, SAE, and CNN-SAE methods in session-to-session classification task. Here, it is shown that anchored-STFT + Skip-Net-GNAA yielded the highest average accuracy value of 78.0 % compared to the other methods. It indicates that proposed method provided 2.9 % higher average accuracy with respect to CNN-SAE method, whereas it provided 5.6 % and 7.7 % improvement in average accuracy with respect to CNN and SAE methods, respectively.

**Supplementary Table 10** shows that, anchored-STFT + Skip-Net-GNAA outperformed CNN-SAE, CNN and, SAE for 6 out of 9 subjects.

***Supplementary Table 10:*** *Comparison of accuracy results generated by CNN, SAE, CNN-SAE [15] and anchored-STFT + Skip-Net-GNAA for session-to-session classification task (trained on 01T and 02T sessions and evaluated on 03T session) of dataset 2b from BCI competition IV.*

| **Subjects** | CNN | SAE | CNN-SAE | anchored-STFT + Skip-Net-GNAA (epsilon = 0.01) |
|---|---|---|---|---|
| S1 | 76.3 | 57.5 | 78.1 | 76.9 |
| S2 | 60.0 | 58.1 | 63.1 | 55.6 |
| S3 | 56.3 | 50.6 | 60.6 | 54.4 |
| S4 | 95.6 | 94.4 | 95.6 | **97.5** |
| S5 | 79.4 | 75.0 | 78.1 | **88.8** |
| S6 | 65.6 | 67.5 | 73.8 | **74.4** |
| S7 | 65.6 | 76.2 | 70.0 | **81.9** |
| S8 | 70.6 | 75.6 | 71.3 | **85.6** |
| S9 | 82.5 | 78.1 | 85.0 | **86.9** |
| Average | 72.4 | 70.3 | 75.1 | **78.0** |

### S5.2 Maximum Kappa value comparison

This analysis shows the comparison of performance of best kappa values of anchored-STFT + Skip-Net-GNAA with some other methods [15], [40], [41], and [42] that provided their best kappa values for dataset 2b of BCI competition IV. It is shown in **Supplementary Table 11** that the average of the best kappa value of our method is higher than all the other methods. Our method outperformed DDFBS [40] and Bi-Spectrum [42] for 6 out of 9 subjects, whereas it outperformed CNN-SAE for 5 out of 9 subjects, whereas it outperformed and RQNN [41] for 4 out of 9 subjects.

***Supplementary Table 11:*** *Comparison of best kappa values of anchored-STFT + Skip-Net-GNAA, CNN-SAE [15], DDFBS [40], Bi-Spectrum [42] and RQNN [41].*

| **Best kappa values** | | | | | |
|---|---|---|---|---|---|
| Subjects | CNN-SAE | DDFBS | Bi-Spectrum | RQNN | anchored-STFT + Skip-Net-GNAA |
| S1 | 0.738 | 0.710 | 0.600 | 0.640 | **0.758** |
| S2 | 0.458 | 0.310 | 0.310 | **0.590** | 0.442 |
| S3 | **0.845** | 0.750 | 0.300 | 0.650 | 0.640 |
| S4 | **1.000** | 0.470 | 0.980 | 0.990 | 0.950 |
| S5 | 0.750 | 0.190 | 0.660 | 0.460 | **0.900** |
| S6 | 0.796 | 0.200 | 0.610 | 0.510 | **0.820** |
| S7 | 0.699 | 0.780 | 0.750 | **0.810** | 0.722 |
| S8 | 0.751 | 0.770 | **0.800** | **0.800** | 0.576 |
| S9 | 0.550 | 0.730 | 0.760 | 0.770 | **0.832** |
| Average | 0.732 | 0.546 | 0.641 | 0.691 | **0.737** |
